# Supplementary material for: Extensive pedigrees reveal the social organization of a Neolithic community
Source: Nature. 2023 Jul 26;620(7974):600–6. doi: 10.1038/s41586-023-06350-8 (PMC10432279; doi:10.1038/s41586-023-06350-8)
Supplement: Supplementary file 1 — Supplementary Notes 1–14.3, Supplementary Tables 26–28, Supplementary Figs. 1 and 2 and Supplementary references. [file 41586_2023_6350_MOESM1_ESM.pdf]

nature portfolio

https://doi.org/10.1038/s41586-023-06350-8

Supplementary information

Extensive pedigrees reveal the social organization of a Neolithic community

In the format provided by the authors and unedited

Nature Supplementary Materials

Extensive pedigrees reveal the social organisation of a Neolithic community

Authors

Maïté Rivollat, Adam Benjamin Rohrlach, Harald Ringbauer, Ainash Childebayeva, Fanny Mendisco, Rodrigo Barquera, András Szolek, Mélie Le Roy, Heidi Colleran, Jonathan Tuke, Franziska Aron, Marie-Hélène Pemonge, Ellen Späth, Philippe Télouk, Léonie Rey, Gwenaëlle Goude, Vincent Balter, Johannes Krause, Stéphane Rottier, Marie-France Deguilloux, Wolfgang Haak

This PDF file includes:

Supplementary Notes
p.1. **Supplementary Note 1.** Regional overview and archaeological context of the Gurgy site
p.6. **Supplementary Note 2.** Biological relatedness and pedigree reconstruction
p.16. **Supplementary Note 3.** Imputation and IBD sharing analysis
p.20. **Supplementary Note 4.** Runs of Homozygosity
p.22. **Supplementary Note 5.** Within-group diversity
p.24. **Supplementary Note 6.** Mitochondrial haplogroup analysis
p.26. **Supplementary Note 7.** Y chromosome haplogroup analysis
p.27. **Supplementary Note 8.** Human Leukocyte Antigens (HLA)
p.29. **Supplementary Note 9.** Analysis of variants associated with phenotypic traits
p.30. **Supplementary Note 10.** Population genetic analyses
p.32. **Supplementary Note 11.** Strontium isotope analysis on tooth enamel and inter/intra individual mobility assessment
p.37. **Supplementary Note 12.** Geospatial analysis
p.40. **Supplementary Note 13.** Bayesian modelling of radiocarbon dates
p.43. **Supplementary Note 14.** Interpretation of the Gurgy necropolis and inferences on the settlement
Supplementary Note 14.1. Social inferences
Supplementary Note 14.2. Demographic inferences
Supplementary Note 14.3. Local mobility inferences from multiple lines of evidence
p.53. **References**

Supplementary Note 1. Regional overview and archaeological context of the Gurgy site
Maïté Rivollat, Stéphane Rottier

Regional context

The first communities of farmers arrived in the northern half of modern-day France around 5200-5000 cal BCE, during the early phases of the Neolithic diffusion[1]. After the initial expansion period, groups tended to segment into several cultural entities, which stabilised during the Middle Neolithic, leading to smaller territories, which were sometimes in competition with each other[2].

Gurgy 'les Noisats' is located in a small region, the Auxerrois, which is part of the Paris Basin and touches the small mountainous massif of Morvan, within the Yonne valley that constitutes a major north-south axis in the local topography. The southernmost settlements from the local Linear Pottery (LBK)-derived culture *Rubané récent du Bassin parisien* (RRBP) and the later Villeneuve-Saint-Germain (VSG) culture in the Paris Basin were excavated in this region. Little evidence of funerary practices from the end of the early Neolithic have been discovered in this area, but what has been found show archaeological features consistent with the wider RRBP area[3,4], such as burials in niches, orientation of the body and types of grave goods.

With the transition to the Middle Neolithic, the complexity of the funerary practices increases in the Paris Basin, and more specifically in the Yonne Valley (Figure Supplementary Note 1). The Cerny culture[5], partly derived from the VSG and representing the final phase of the LBK, is present in the region from about 4700 BCE, and is known for its characteristic monumental funerary structures: the so-called *Structures de Type Passy* (STP, ~4700-4300 cal BCE). On the western side of the Yonne River, along the Essonne River, a group of sites associated with the Cerny culture represents another form of funerary type. Here, some individuals were buried under a large slab (Orville 'les Fiefs' and Malesherbes 'les Marsaules', ~4700-4350 cal BCE), while others from the same site were buried in simple burials[6,7]. These sites are named 'Malesherbes type' and at least four others have been detected in the area, but have not been excavated[8] (Figure Supplementary Note 1). The influence of exogenous cultures is well-established, notably of the Chasseen culture from the south and represented in the graveyard Monéteau 'Macherin', and archaeological artefacts from the southern sphere are spread all over the Paris Basin[9,10]. Another element showing southern influences is the Chamblandes-like rigid containers, found in Gurgy, but also in Monéteau, and was mainly present in the Alps and in central France[11].

Overall, archaeological cultures and distinct funerary practices are not in agreement, as different burial types or body positions, for instance, were found within the same archaeological group. The spatial boundaries are also not strict, and the different archaeological entities are rather permeable. Most of the funerary sites located in the Paris Basin *sensu stricto* have STPs implanted[12], of which there are around twenty in the region (Figure Supplementary Note 1). These long and sometimes massive mounds follow a specific set up which, however, varies from one site to another. Only a few individuals are buried in the mounds, obviously not representing the entire community, but rather select individuals. Female and male adults and subadults are found buried in the same monument. When several monuments are grouped together, in some cases (e. g. Balloy) males and females separated from each other, each in one monument[13]. In fact, at some sites, these monuments are found in pairs, one of which is female-specific, and the other is male-specific. Pairs of male monuments can also be found, organized around a central individual, buried with specific grave goods such as the 'Eiffel Tower', made from a cervid scapula and exclusively associated with males. Even if grave goods are generally scarce in STP sites, these express a

strong association to hunting and the "wild world"[14]. Indeed, arrowheads and adornment and tools made from bone material from wild species are commonly found in the graves. This does not correspond to an economy based on hunting practices, however, as the subsistence of the Cerny culture is clearly based on agriculture, but the 'persona/role of the hunter' is nevertheless expressed in these selected graves of the STPs[15].

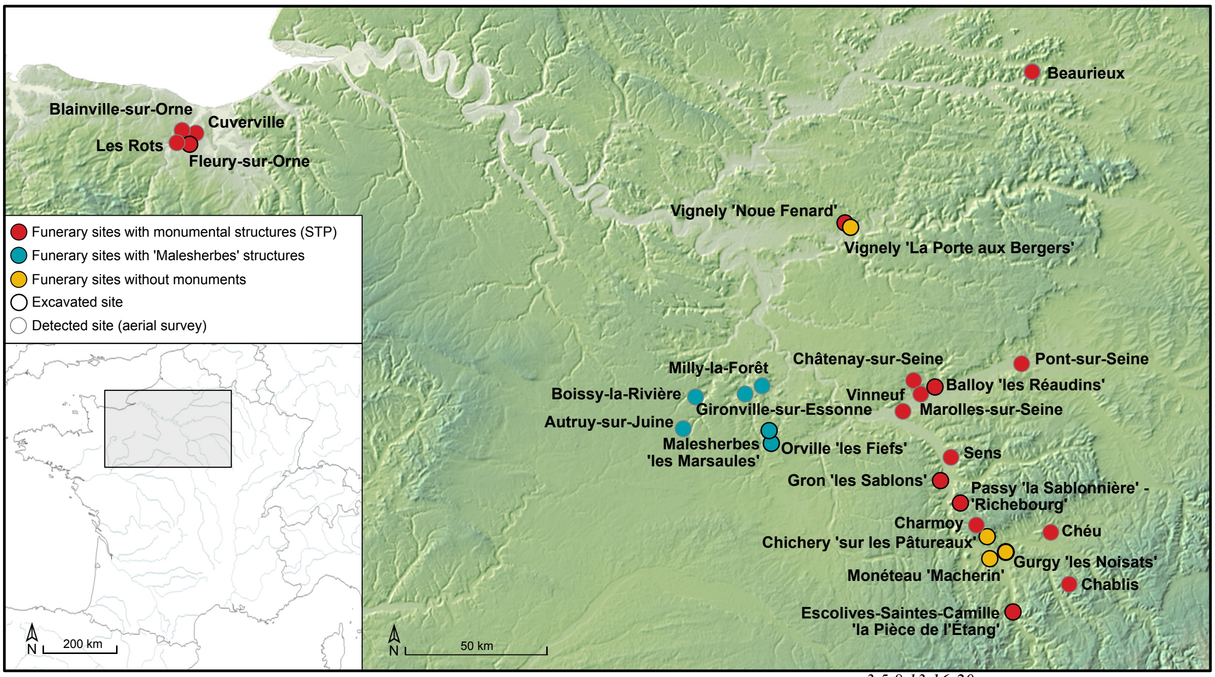

***Figure Supplementary Note 1. Geographic distribution** of funerary sites[3,5,8,13,16-20] during the Cerny period in the extended Paris Basin (background map ©PachaCartographie, https://www.pacha-cartographe.fr/fonds-de-carte/). Map of France created with QGIS software (v.3.30) under the Sharealike license (https://creativecommons.org/licenses/by-sa/3.0/).*

The Cerny culture expands beyond the Paris Basin *sensu stricto* to the north, where aerial surveys have documented a group of four STP sites in Normandy (Blainville-sur-Orne, Les Rots, Cuverville, Fleury-sur-Orne)[19,21] (Figure Supplementary Note 1). Of these, Fleury-sur-Orne is the only site that has been excavated extensively and for which genetic data is available[22]. It is unclear how representative this site was for the region, but the site of Fleury shows differences when compared to the STP from the Paris Basin. The monuments at Fleury are earthen long barrows, some measuring up to 300 m in length, making them the longest in Europe. They were built for a single individual, or rarely for two, highlighting the very specific status of the individuals buried in them. Using genetic data from these ancient individuals, a recent study on Fleury[22] showed that: (a) almost all the individuals were males (13/14), (b) the pairs of individuals buried in the same monuments/graves were father and son, and (c) that there was no close biological relationship between individuals from the different monuments. The combined data and documented father–son line of descent suggest a transmission of sociopolitical authority through males.

Concerning the specific regional context of STP sites in the Paris Basin, only a few funerary sites do not fit to this monumental pattern: Chichery 'sur les Pâtureaux' (~4700-4300 cal BCE)[17], Vignely 'La Porte aux Bergers' (~4800-4400 cal BCE)[18,20], Monéteau 'Macherin' (~4500-4000 cal BCE)[16], and Gurgy 'les Noisats'[3] (Figure Supplementary Note 1).

The graveyard Chichery 'sur les Pâtureaux'[17] contains 15 individuals in 13 graves, including adult and subadult females and males. Except for one, all of the graves belong to

the Balloy type, *i.e.*, individuals were laid down on their back, a position usually associated with the Cerny monumental funerary sites in the Paris Basin. Although monuments are absent from this graveyard, these graves are associated with the Cerny culture on the basis of the grave type and the archaeological context.

Vignely 'La Porte aux Bergers' ranges from the Early to the Middle Neolithic[18] and included 43 individuals. Twenty-seven of these individuals, buried in 22 graves, were ascribed to the Cerny culture, with 71% of the deceased laid down on their back, with the others in a flexed position on their left side. There were also no signs of any associated monuments. It is possible that the graveyard extends beyond the currently excavated area and that the record is thus incomplete. However, subadult and adult females and males were identified here[20].

Monéteau 'Macherin'[16] is a site located about 3 km far from Gurgy 'les Noisats' on the opposite side of de Yonne river. Ranging from the Early to the Late Neolithic, one enclosure and two groups of burials date to the Middle Neolithic. The group of 16 burials located in the North is exhaustively excavated and includes 17 individuals. Burials are aligned in an area that is about six meters wide and 40 meters long, but no monument is associated with the burials. Individuals were buried in a left-side flexed position, within perishable material containers. 100 meters to the south is the second group of burials, represented by four graves and six individuals, but the excavation in this aera was not exhaustive. Here, too, no monument is associated with the graves. A container made of perishable material was found in only one grave, and graves from this group are less homogenous overall. The northern and southern groups are contemporaneous, and the grave goods (pottery and arrowheads) were ascribed to the *Chasséen* culture, which is predominantly distributed in southern France. The perishable containers and the left-side flexed position of the bodies are similar to the Chamblandes cists in the Lemanic Basin and in the Massif Central. Beside these clear southern influences, some flint tools were attributed to the Cerny culture.

In this complex regional picture, Gurgy 'les Noisats' represents one of four graveyards without monuments, and stands out due to the exceptionally great number of graves[3].

<u>The Gurgy site</u>

Direct radiocarbon dates from human remains from Gurgy 'les Noisats' range between 5000 and 4000 BCE, but the most intensive occupation period ranges from 4900 to 4500 BCE, which corresponds to the end of the early Neolithic, and the beginning of the Middle Neolithic. The complete excavation of the graveyard took place between 2004 and 2007 under the direction of S. Rottier[3,23].

A total of 134 pits were excavated at the site, uncovering 128 individuals, which makes Gurgy the biggest funerary site for these periods in the Paris Basin discovered so far[24]. The concentration of the graves is confined within a perimeter of 500 m^2, without many overlaps, which implies that the group knew of the location of the graves, or that there were surface marks at the time. Experimental work with the local sediment at the site showed that the digging of a pit, once refilled with the same sediment, leaves an indentation on the ground, allowing the people of Gurgy to visualize the location of each grave and to avoid the overlaps[25].

Most uncovered pits were single graves but there were some cases of double burials. In Supplementary table 21, we include information about single and double burials, in which individuals were buried simultaneously in the same pit (for instance GLN207A and GLN207B) or were buried one after the other in the same pit (for instance GLN221A and GLN221B). The site includes a range of diverse grave types: burials in pits without construction, burials using a rigid coffin of perishable/organic matter, likely wood, burials in

pits with more elaborate architecture, also not preserved, and burials in niches[23]. The pit sizes vary from 0.71m to 2.83m according to the grave type, and only two are substantially (approximately two to three times) larger than the rest: GLN221B (2.80x1.35m) and GLN237A (2.83x0.81m). The diversity in grave types suggests cultural influences from various different regions. Rigid containers made of perishable matter are similar to those found in Monéteau 'Macherin'[16]. In Gurgy, as in Monéteau, these burials in rigid containers echo those found in regions like modern-day Switzerland or the Massif Central, and are attributed to the Chamblandes phenomenon[11]. By contrast, burials in niches were common during the time of the RRBP, but those in Gurgy do not strictly follow the standard orientation of the RRBP[4], both for the graves and for the niches[3].

Grave goods, notably pottery and flint tools, are scarce and randomly distributed across the cemetery. The elements of adornment, which are also rare, are remarkably diverse and include shells (e.g. scallop), perforated animal teeth (e.g. beaver), and limestone beads[3]. Overall, the observed spectrum of grave goods does not allow for a clear attribution of Gurgy to a single Neolithic culture. For example, the presence of ochre in 14 burials is a characteristic feature of the western LBK, while some shells, specifically one scallop, points to the Mediterranean sphere (Supplementary table 21, Extended Data Fig. 3c). Despite being the biggest graveyard in the Paris Basin, the site Gurgy 'les Noisats' does not match the culturally encoded funerary practices known from the region at the time, and thus represents an exception with respect to the local archaeological record.

Gurgy individuals

An anthropological investigation was conducted on all of the 128 excavated individuals. Morphological age classes for adults were assigned using the morphology of the iliac sacro-pelvic surface[26]. For subadults, tooth growth and bone maturation were used[27,28]. Osteological sex was estimated for adult and young adult individuals using the pelvis bone[29,30], and, if not available, by applying a secondary diagnosis method[31]. 66 adult and 62 subadult individuals were identified, and from these, the genetic sex of 30 males and 20 females could be assigned, leaving 23 individuals remaining undetermined (Supplementary table 1). It should be noted here that the individual GLN270A, previously published as undetermined[32], has been revised in this study. Indeed, this individual could be determined to be female during the excavation in the field by standard anthropological sex determination and documented as such in the field notes. However, the pelvis bones broke during sampling/further handling, and therefore this diagnosis could not be confirmed during the laboratory analysis and was kept as undetermined. We refer the original field notes for this study.

Applying archaeothanatological methods[33], bodies were proved to have decomposed in an empty space, demonstrating the presence of a rigid container or coffin. Bodies were mainly placed in a left-side flexed position, with some exceptions (more or less flexed, lying on their back). The main orientation of the bodies is north-south (Supplementary table 21).

Since the excavation, several studies were conducted on the human remains, specifically to look for a rationale explaining the structure of the site. The variation of the internal tooth structures such as enamel thickness, tissue proportions, and the three-dimensional shape of enamel-dentin junction was investigated for 20 individuals at the site level, and variations between individuals buried in graves with different types were detected[34].

Stable isotope analysis of bone collagen ($\delta^{13}C$, $\delta^{15}N$, $\delta^{34}S$) was performed on Gurgy individuals to study diet practices and mobility[32,35,36] (Supplementary table 24). The archaeozoological record reported the consumption of predominantly cattle, but data from

Gurgy, similar to other local sites, also suggest a mixed protein consumption of cattle and pig, possibly complemented with some freshwater resources[32]. A great homogeneity was found within the Gurgy individuals. However, some differences between males and females were identified both for diet[35] and mobility[36].

Previous aDNA analyses reported mitochondrial haplotypes from 55 individuals (based on sequences of the hypervariable region 1), which showed a variability matching the expectations for Neolithic periods in western Europe, and suggested a connection with the Mediterranean sphere[37,38]. The analysis of genome-wide data confirmed the genetic homogeneity of prehistoric individuals from western Europe during the Middle Neolithic, and provided evidence for admixture with local hunter-gatherers[39].

An integrative study combining the mitochondrial results with archaeological data was also performed in order to understand the organisation of the site and to test whether any archaeological feature (grave goods, grave type, body orientation, etc.) could be linked to specific maternal lineages[40]. This work showed that no correlation was observed between mitochondrial lineages and archaeological data, and hence is not helping to understand the structure of the graveyard.

Additionally, on the basis of positive indication of a Hepatitis B virus (HBV) infection, two individuals (GLN201 and GLN258) formed part of a larger survey study focusing on the evolutionary history of HBV[41]. Given the nature of the horizontal and vertical transmission mechanisms of HBV, it is of interest to note that only two positive cases were detected among the studied individuals. Since both individuals were subadults, it is reasonable to expect that we would also identify the infection in their biological mothers. However, we found no evidence of transmission in the case of the pair GLN258/GLN249, while the mother of GLN201 was not found among the studied individuals.

Supplementary Note 2. Biological relatedness and pedigree reconstruction
Maïté Rivollat, Adam Benjamin Rohrlach, Marie-France Deguilloux, Wolfgang Haak

Methods

READ

We assessed the degree of genetic relatedness using autosomal data from the complete dataset of 94 individuals by applying different approaches. We used the software READ[42] that calculates and averages the mismatch rate, denoted p_0, for non-overlapping windows of 1M base pairs (bp) across the whole genome on pseudo-haploid genotype calls with randomly called SNP sites on the 1240k set (Supplementary table 8). The median value of p_0 for pairs was estimated to be 0.242, which is interpreted as the expected pairwise mismatch rate for two unrelated individuals. Outliers with lower mismatch rates, *i.e.*, lower values of p_0, indicate more closely genetically related individuals, where the lowest values are expected for 1st-degree relatives (given that there are no monozygotic twins among the studied individuals. When correcting for the median expected mismatch rate for unrelated individuals, thresholds for degrees of relatedness are calculated by READ and are set to 0.151 between identical libraries and 1st-degree related individuals, 0.196 between 1st-degree and 2nd-degree related individuals and 0.219 between 2nd-degree and unrelated individuals (Supplementary table 8). READ also provides standard errors to allow for uncertainty caused by poorer-coverage individuals.

lcMLkin

In order to confirm the findings from READ, we used the software lcMLkin, which estimates the probability of identity-by-descent (IBD) from genotype likelihoods[43]. lcMLkin aims to estimate the coefficient of relatedness *r,* the proportion of the genome common to two individuals due to direct kinship. For example, *r* equals 1⁄2 in the case of parent-offspring or siblings, 1/4 for 2nd-degree related individuals, in non-inbred pedigrees. lcMlkin estimates *r* by estimating three parameters: k_0, k_1 and k_2. These probabilities measure the relatedness between individuals, as they represent, respectively, the probability that none, one or two alleles, are shared via IBD between the pair of individuals. Note that $k_0 + k_1 + k_2 = 1$, and that $r = k_1/2 + k_2$. While $r = 1/2$ for both of the 1st-degree relationships between parent/child and full siblings, the probabilities k_0, k_1 and k_2 differ, and are (1/4,1/2,1/4) for full siblings, and (0,1,0) for parent/child[44]. Inspection of the estimated values for k_0, k_1 and k_2 then allows us to distinguish between both categories of 1st-degree relationships. As the method is applied to genotype likelihoods, low-coverage data can give unreliable results, and so we therefore restricted to pairs that share at least 10,000 SNPs. We also set the thinning parameter to 10,000 to thin SNPs so that they are at least 10,000 sites apart. Results are shown in Supplementary table 9 and the coefficient of relatedness *r* and the probability k_0 are plotted in Extended Data Fig. 1a. The Extended Data Fig. 1a allows us to visualize the two clusters of parents-offspring and siblings amongst 1st-degree related individuals.

PMR-window approach

To investigate the spatial distribution of the PMR for 1st-degree relatives, and how these might differ for parent-child and sibling relationships, we plotted the PMR along the genome in windows of width 1 Megabase. As stated above, the probabilities of sharing 0, 1 or 2 alleles is different for full-siblings and parent-child relationships, (1/4,1/2,1/4) and (0,1,0) respectively. Hence it should be visible that the windowed estimate of PMR for parent-child relationships is stable around the average PMR, whereas the windowed estimate for full-siblings should be more variable. Thus, to differentiate between parent-child and full-

sibling 1st-degree relationships, we visually compare the windowed estimate of PMR to the chromosomal average and the genome-wide average (Extended Data Fig. 1c). However, when one, or both, of the samples are low-coverage, it can be difficult to differentiate between variability in the windowed estimate of the PMR due to coverage, or due to a difference in the nature of the 1st-degree relationship.

Binomial-PMR approach

In the case of ambiguous results given by lcMLkin and READ, we developed a new approach called BREADR (v. 1.0.1)[45] to estimating the PMR for a pair of individuals based on an assumption of a binomial distribution for the PMR, for pseudo-haploid data, available at https://github.com/jonotuke/BREADR. Here, for individuals *i* and *j*, we thinned the data such that all sites were at least 200K bases apart to best satisfy the assumption of independence. We did so by taking all of the overlapping sites, and their relative positions. We started with chromosome 1, where we took the first overlapping site, retained it, then took the next site that was a minimum of 200K sites along the chromosome, and retained it, etc. We repeat this until we had no overlapping sites left. We then repeat this “per chromosome” process for chromosomes 2 through to 22. In this way we found a likely optimally large set of thinned, overlapping SNPs.

We then calculated the number of mismatching base calls, denoted $X_{i,j}$, out of the possible $N_{i,j}$ overlapping sites. We then calculated the “thinned” PMR, $p_{i,j} = X_{i,j}/N_{i,j}$. Using the logic of READ, we calculate the median PMR, denoted $\hat{p}_u$, and define the expected PMR for a *k*-degree related pair as $\hat{p}_k = (1 - 1/2^{k-1})\hat{p}_u$, for $k = 0,1,2$.

We then find the likelihood of the observed PMR for each value of *k* using the probability density function of a binomial distribution, denoted

$$P(X_{i,j}|K = k) = \binom{X_{i,j}}{N_{i,j}} \hat{p}_k{}^{X_{i,j}} (1 - \hat{p}_k)^{N_{i,j}-X_{i,j}}$$

Note that we define two individuals as “unrelated” if they are more distantly than 2nd-degree related, i.e. 3rd-degree or more.

$$P(X_{i,j}|K \geq 3) = \sum_{k=3}^{\infty} \binom{X_{i,j}}{N_{i,j}} \hat{p}_k{}^{X_{i,j}} (1 - \hat{p}_k)^{N_{i,j}-X_{i,j}} f_\lambda(k),$$

where $f_\lambda(k)$ is the 3-truncated Poisson distribution of the form

$$f_\lambda(k) = \frac{\frac{\lambda^k e^{-\lambda}}{k!}}{\sum_{j=0}^{2} \frac{\lambda^j e^{-\lambda}}{j!}},$$

chosen with $\lambda = 10$ as it represents well the un-likelihood of individuals always being closely related once they are more than second-degree related, but also captures the diminishing probabilities of being too distantly-related due to the finite size of populations.

We can then calculate the posterior probability of the individuals *i* and *j* sharing a *k* degree relationship as

$$P(K = k|X_{i,j}) = \frac{P(X_{i,j}|K = k)P(K = k)}{\left[\sum_{k=0}^{2} P(X_{i,j}|K = k)\right] + P(X_{i,j}|K \geq 3)},$$

for which the denominator is equal to one by construction. We note that a formulation of the posterior probabilities with P(K=k) = ¼ inherently assumes that all degrees of relatedness are

equally likely, but it is not clear what these probabilities should be, and so we used this uninformative prior.

All cases involving 1st, 2nd and problematic 2nd-degree/unrelated relationships are plotted individually and provided at https://doi.org/10.5281/zenodo.7224898.

Tree reconstruction

To reconstruct the pedigree trees, we started by reconstructing small families linked only by 1st-degree relatedness according to the results of READ and lcMLkin (Supplementary tables 8 and 9). We combined these with maternal and paternal lineages to restrict the possible topologies to consistent trees, and with the age-at-death of the individuals to rule out impossible parent-offspring relationships (i.e., a young subadult individual cannot be a parent). With this first step we were able to reconstruct 17 small family trees, consisting of between 2 and 17 individuals.

We then used the 2nd-degree relationships inferred by READ (Supplementary table 8) to connect together these different small family trees together. Note that 2nd-degree related individuals could be grandparents/grandchildren, half-siblings or uncle-aunt/nephew-niece. Consistencies between these small family trees allowed us to double-check every connection.

We then applied the PMR-window and the binomial-PMR approaches to clarify potentially inconsistent results.

Reconstruction of pedigrees involving 1st-degree related individuals

- GLN206 and GLN208 are two children 1st-degree related as siblings (Supplementary tables 8 and 9, Figure Supplementary Note 2. I).

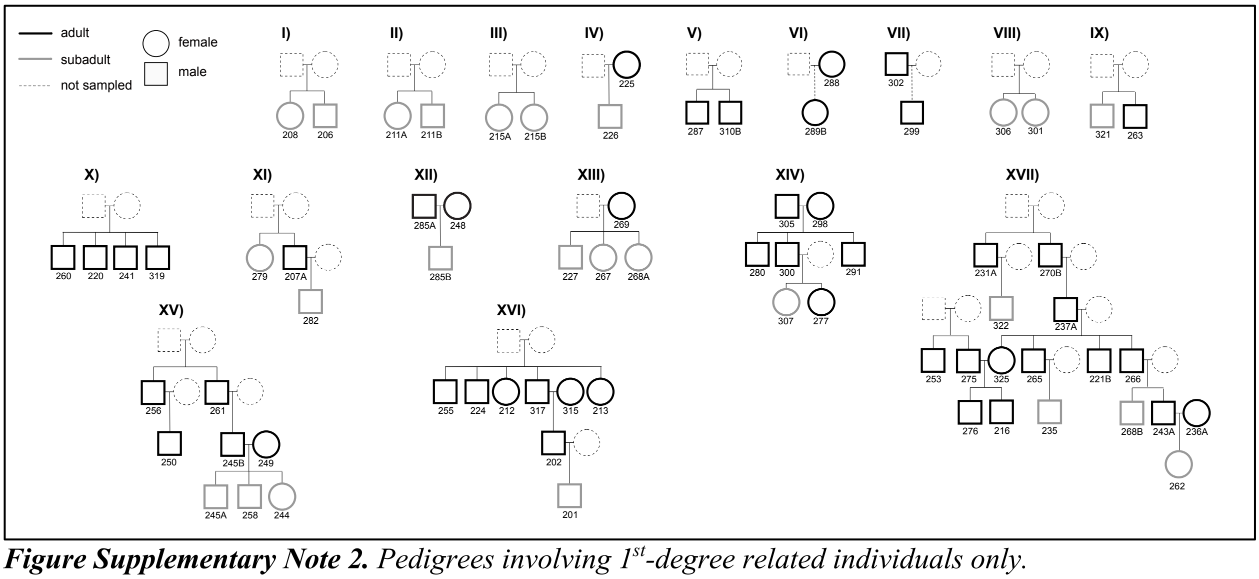

Figure Supplementary Note 2. *Pedigrees involving 1st-degree related individuals only.*

- GLN211A and GLN211B are two children 1st-degree related as siblings (Supplementary tables 8 and 9, Figure Supplementary Note 2. II).

- GLN215A and GLN215B are two female children who are siblings (Supplementary tables 8 and 9, Figure Supplementary Note 2. III).

- GLN225 and GLN226 are two individuals who are 1st-degree related as parent-offspring. Given the young age of GLN226 (1-3 years old) and the shared mitochondrial haplogroup, they must be mother and son (Supplementary tables 8 and 9, Figure Supplementary Note 2. IV).

- GLN287 and GLN310B are two adult males, 1st-degree related as siblings (Supplementary tables 8 and 9, Figure Supplementary Note 2. V).

- GLN289B and GLN288 are two female adults, 1st-degree related (Supplementary table 8, Figure Supplementary Note 2. VI). At this stage, the low coverage of GLN289B does not allow to specify if they are siblings or mother-daughter.

- GLN299 and GLN302 are father and son, but as both are male adults, we cannot determine who is the father and who is the son (Supplementary tables 8 and 9, Figure Supplementary Note 2. VII). The dotted line indicates the uncertainty.

- GLN301 and GLN306 are two female children who are siblings (Supplementary tables 8 and 9, Figure Supplementary Note 2. VIII).

- GLN321 and GLN263 are one infant and one adult male and are siblings (Supplementary tables 8 and 9, Figure Supplementary Note 2. IX).

- GLN220, GLN241, GLN260 and GLN319 are four adult male siblings (Supplementary tables 8 and 9, Figure Supplementary Note 2. X).

- GLN207A and GLN279 have a READ p_0 value at the threshold between 1st and 2nd degree, but lcMLkin results clearly show a 1st-degree sibling relationship (Supplementary tables 8 and 9, Figure Supplementary Note 2. XI). GLN282 has a parent-offspring connection to GLN207A but not to GLN279 with who he is 2nd-degree related. He therefore can only be the son of GLN207A (Figure Supplementary Note 2. XI).

- GLN285B is a male child who shares a 1st-degree relationship with GLN285A and GLN248 who are not related to each other (Supplementary tables 8 and 9). The connection given by lcMLkin for GLN285B with GLN285A is the only inconsistent one (red dot within the blue cluster on Extended Data Fig. 1a). Given the PMR-window plot (Extended Data Fig. 1c) and the absence of 2nd-degree relationships between GLN285B and both GLN245B and GLN261 (see below), GLN285B cannot be sibling with GLN285A. Hence, GLN285A and GLN248 are the parents of GLN285B (Figure Supplementary Note 2. XII).

- GLN269 has a parent-offspring relationship with GLN267, GLN227 and GLN268A who are all siblings (Supplementary tables 8 and 9). GLN269 must be their mother (Figure Supplementary Note 2. XIII).

- GLN305 and GLN298 have a parent-offspring relationship with GLN280, GLN291 and GLN300, and are not related to each other. GLN280, GLN291 and GLN300 are siblings. GLN307 and GLN277 are siblings, and both 1st-degree related to GLN300 in a parent-offspring relationship. Their 2nd-degree relationship with GLN305 and GLN298, and

with GLN280 and GLN291, clearly make them the offspring of GLN300 (Supplementary tables 8 and 9, Figure Supplementary Note 2. XIV).

- GLN256 and GLN261 are two adult males who are 1st-degree related as siblings. GLN250 is 1st-degree related to GLN256 with a parent-offspring relationship, and as he shares a 2nd-degree relationship with GLN261, he can only be the son of GLN256 and the nephew of GLN261. Similarly, GLN245B is 1st-degree related to GLN261 but shares a 2nd-degree relationship with GLN256 and is therefore the son of GLN261 and nephew of GLN256. GLN245B and GLN249 are not related, and they have a parent-offspring relationship with GLN245A, GLN258 and GLN244 who are siblings, and who also have a 2nd-degree relationship with GLN261 who must therefore be their grandfather (Supplementary tables 8 and 9, Figure Supplementary Note 2. XV).

- GLN317, GLN212, GLN213, GLN255 and GLN224 all share a 1st-degree sibling relationship. GLN317 and GLN315 are not related, and both have a parent-offspring relationship with GLN202 who is their son. GLN202 indicated a 2nd-degree relationship with all of the siblings of his father GLN317. GLN202 shares a parent-offspring relationship with GLN201 who also shares a 2nd-degree relationship with GLN317 and GLN315. He is therefore the son of GLN202 (Supplementary tables 8 and 9, Figure Supplementary Note 2. XVI).

- GLN237A has a parent-offspring relationship with GLN325, GLN265, GLN221B and GLN266, who are all siblings. GLN237A can only be their father (Supplementary tables 8 and 9, Figure Supplementary Note 2. XVII).
  GLN325 and GLN275 are not related to each other, and they share a parent-offspring relationship with GLN216 and GLN276, who are themselves siblings. Both GLN216 and GLN276 share a 2nd-degree relationship with the siblings of GLN325, which is expected as they are their uncles, as well as with GLN237A who is their grandfather. Their father GLN275 has a brother GLN253 who also shares an uncle-nephew relationship with both GLN216 and GLN276 (Supplementary tables 8 and 9, Figure Supplementary Note 2. XVII).
  GLN265 is the father of GLN235 who shares a 2nd-degree relationship with all of the siblings of GLN265, as they are his uncles and aunt, and with GLN237A, as he is his grandfather (Supplementary tables 8 and 9, Figure Supplementary Note 2. XVII).

- GLN266 is the father of the siblings GLN268B and GLN243A, who both also share a 2nd-degree relationship with all of the siblings of GLN266, as they are their uncles and aunt, and with GLN237A as their grandfather. However, given the low coverage of GLN268B, some 2nd degrees are missing in the READ results. By looking at the binomial-PMR distributions, the posterior probabilities of being 2nd degree with the siblings of GLN266 are variable (with GLN325: 7%, GLN262: 12%, GLN221B: 47%, GLN265: 88%), and it stands at 98% with the grandfather GLN237A (see binomial-PMR distribution plots). However, given the configuration of the tree and the other connections, this is the only possible scenario (Supplementary tables 8 and 9, Figure Supplementary Note 2. XVII).

- GLN243A is unrelated to GLN236A and both share a 1st-degree relationship with their daughter GLN262 (Supplementary tables 8 and 9, Figure Supplementary Note 2. XVII).

- GLN270B, who had very low coverage, and for whom we could not assess the mitochondrial haplogroup, shares a 1st-degree relationship with GLN237A, but we cannot determine from the genetic data if it is a sibling or parent-offspring relationship. GLN270B shares a 2nd-degree relationship with the offspring of GLN237A, as expected if he is their grandfather, or their uncle (Supplementary tables 8 and 9, Figure Supplementary Note 2. XVII).
  GLN270B also shares a 1st-degree relationship with GLN231A, who shares a 2nd-degree relationship with GLN237A, which is not possible if GLN237A and GLN270B were brothers (Supplementary tables 8 and 9). The only possibility is therefore that GLN270B is the father of GLN237A and GLN231A is the brother or father of GLN270B, and uncle or grandfather of GLN237A. Unfortunately, we cannot resolve the relationship between GLN270B and GLN231A on the basis of the available data (Supplementary tables 8 and 9). As the PMR-window plot suggests a sibling relationship (Extended Data Fig. 1c), we nevertheless chose to keep this reconstruction in the pedigrees. GLN231A shares a parent-offspring connection with GLN322 who is a child. He cannot be the father of GLN231A, and he shares a 2nd-degree connection GLN270B, who can be his uncle if GLN270B is the brother of GLN231A, or his half-brother if GLN270B is the son of GLN231A (Supplementary tables 8 and 9, Figure Supplementary Note 2. XVII).

Connections between the nuclear families with 2nd-degree relationships

Pedigree A

- The adult male GLN320 shares a 2nd-degree relationship with GLN231A and GLN270B (Supplementary table 8). He could be either their grandfather, their uncle, or their nephew, all through the paternal line. The absence of other related individuals does not allow us to decide between these options, and the relationship is thus shown as uncertain in Figure 1a.

- The siblings GLN215A and GLN215B share a 2nd-degree relationship only with GLN276 (Supplementary table 8). They cannot be his half-siblings because they would also be 2nd-degree related to GLN216, the brother of GLN276, and 1st-degree related to one of the parents GLN275 and GLN325. They also cannot be the nieces of GLN276 because they would be 2nd-degree related to GLN216 as nieces, but also to GLN275 and GLN325 as granddaughters. They can only be the granddaughters of GLN276, and we cannot say if it is via the paternal or maternal side, as they do not carry a Y chromosome and their mitochondrial DNA is anyway different from the one carried by GLN276 (Fig. 1a).

- The siblings GLN227, GLN267 and GLN268A share a 2nd-degree relationship with GLN266, GLN268B and GLN243A (Supplementary table 8). The only possibility of a 2nd-degree relationship with both a parent and an offspring is if one is the grandchild of the former and the nephew/niece of the latter. GLN227, GLN267 and GLN268A are therefore grandchildren of GLN266, and GLN243A and GLN268B are both their uncles. The relationship can only be via the missing father, as the mother GLN269 does not share a close relationship with either GLN266, GLN243A or GLN268B (Fig. 1a).

- The male individual GLN236B is 2nd-degree related to GLN262, GLN236A and GLN243A (Supplementary table 8). The only possibility is for him to be the nephew of GLN262 and the grandson of GLN236A and GLN243A. As this individual carries a

different mitochondrial haplotype than his grandmother, and the same Y-chromosome lineage as his grandfather, we can assume that the link is made through the missing father (Fig. 1a).

- Similarly, the female individual GLN214 is 2nd-degree related to GLN262, GLN236A and GLN243A (Supplementary table 8). She can only be the niece of GLN262 and the granddaughter of GLN236A and GLN243A. As she carries a different mitochondrial haplotype than her grandmother, we can also assess that the link is made through her missing father (Fig. 1a).

- GLN223 shares a 2nd-degree relationship with GLN202, GLN315 and GLN317 (Supplementary table 8). She can only be the niece of GLN202 and the granddaughter of GLN315 and GLN317. She does not carry the same mitochondrial haplotype as her grandmother; therefore, the link can only exist through her missing father (Fig. 1a).

- GLN210 is 2nd-degree related to the siblings GLN212, GLN255 and GLN317 (Supplementary table 8). According to the READ results, both pairs GLN210-GLN213 and GLN210-GLN224 show values just below the established threshold (Supplementary table 8). By checking both pairs with our binomial-PMR approach, we can confirm that they are 2nd-degree related, with posterior probabilities of 1 and 0.99 respectively (see binomial-PMR distribution plot). The two explanations for 2nd-degree relationships with siblings are either a half-sibling or an uncle/aunt – nephew/niece connection. If GLN210 was half-sibling with these brothers and sisters via their father, he would also be 2nd-degree related to GLN237A, GLN325, GLN265, GLN221B and GLN266, which is not the case. If it was via their mother, they would all share the same mitochondrial haplotype, which is not the case. Therefore, GLN210 can only be the nephew or the uncle of the siblings GLN212, GLN213, GLN224, GLN255 and GLN317. If he was an uncle via the paternal line, he would be expected to be 1st-degree related to the missing father's siblings GLN221B, GLN265, GLN266 and GLN325, which is not the case. If he was an uncle via the maternal line, he would be expected to share the same mitochondrial haplotype with the missing mother's offspring GLN212, GLN213, GLN224, GLN255, GLN317, which is also not the case. Therefore, he can only be a nephew via the missing father's side, because of non-matching mitochondrial haplotypes with the uncles GLN224, GLN255, and GLN317, and the aunts GLN212 and GLN213 (Fig. 1a).

- Almost all the siblings GLN212, GLN213, GLN224, GLN255 and GLN317 share a 2nd-degree relationship with the siblings GLN325, GLN265, GLN221B and GLN266, as well as to their father GLN237A, according to READ results (Supplementary table 8). A few pairs are categorized as unrelated by READ, but the binomial-PMR distribution confirm the 2nd degrees (GLN213 and GLN221B, 0.91; GLN255 and GLN221B ~1; GLN317 and GLN221B, 0.92; GLN317 and GLN237A, 0.99; see binomial-PMR distribution plot). The only possibility is for them to be nephews/nieces of GLN325, GLN265, GLN221B and 266 and grandchildren of GLN237A. The missing parent, offspring of GLN237A, can only be a male as the siblings GLN212, GLN213, GLN224, GLN255 and GLN317 do not carry the same mitochondrial haplotype than GLN325, GLN265, GLN221B and GLN266 (Fig. 1a).

- GLN204 shares a 2nd-degree relationship with GLN207A and GLN282 (Supplementary table 8). The only possibility is that he is the nephew of GLN282 and grandson of

GLN207A. As he carries a different mitochondrial haplotype than his uncle, we assume a link through the missing father (Fig. 1a).

- GLN206 and GLN208 are 2nd-degree related to GLN207A and GLN282 (Supplementary table 8). Similar to the previous case, they must be nephew and niece of GLN282 and grandchildren of GLN207A. As they carry a different mitochondrial haplotype than their uncle, we assume a link through their missing father (Fig. 1a).

- The siblings GLN207A and GLN279 are 2nd-degree related to the siblings GLN263 and GLN321 (Supplementary table 8). If they were half-siblings connected by their father, they would also be 2nd-degree related to GLN237A, GLN325, GLN265, GLN221B and GLN266, which is not the case. If they were half-siblings connected by their mother, they would all share the same mitochondrial haplogroup, which is not the case. If they were uncle and aunt via the paternal line, they would be expected to be 1st-degree related to the missing father's siblings GLN221B, GLN265, GLN266 and GLN325, which is not the case. If they were uncle and aunt via the maternal line, they would be expected to share the same mitochondrial haplotype with the missing mother's offspring GLN263 and GLN321, which is also not the case. Therefore, GLN207A and GLN279 must be the nephew and niece of the siblings GLN263 and GLN321, via a missing father as they do not share the same mitochondrial haplogroup (Fig. 1a).

- GLN209 is 2nd-degree related to GLN263 and GLN321 (Supplementary table 8). According to the same rationale, he can only be their nephew, via a missing father (Fig. 1a).

- In the same way, GLN257 is 2nd-degree related to GLN263 and GLN321 (Supplementary table 8). He can only be their nephew, via a missing father (Fig. 1a).

- The siblings GLN263 and GLN321 share a 2nd-degree relationship with the siblings GLN325, GLN265, GLN221B and GLN266, as well as to their father GLN237A (Supplementary table 8). The only possibility is that they are the nephews of GLN325, GLN265, GLN221B and GLN266 and grandchildren of GLN237A. The missing parent, the offspring of GLN237A, can only be a male as the siblings GLN263 and GLN321 do not carry the same mitochondrial haplotype as GLN325, GLN265, GLN221B and GLN266 (Fig. 1a).

- GLN232C shares a 2nd-degree relationship only with GLN319 (Supplementary table 8). She cannot be his half-sibling because she would be expected to also be 2nd-degree related to GLN220, GLN241 and GLN260, brothers of GLN319. Given the young age (2-6 years old) of GLN232C, she can also not be his grandmother. She also cannot be the niece or the aunt of GLN319 because she would be expected to also be 2nd-degree related to GLN220, GLN241 and GLN260. She can only be the granddaughter of GLN319, but we cannot distinguish between the paternal or maternal side, as she does not carry a Y chromosome and her mitochondrial haplogroup would not be transmitted by GLN319 (Fig. 1a).

- GLN226 is 2nd-degree related to all siblings GLN220, GLN241, GLN260 and GLN319, with whom his mother GLN225 does not share any genetic relationship (Supplementary table 8). He cannot be their half-sibling via their father because we would also share a 2nd-degree relationship with GLN237A as an uncle and with GLN270B as a grandfather,

which is not the case. He cannot be their uncle via the father because he would be expected to be 1st-degree related to GLN237A, neither via the mother because GLN225 would therefore be 2nd-degree related to the siblings GLN220, GLN241, GLN260 and GLN319 as their grandmother, which is not the case. Thus, GLN226 can only be the nephew of GLN220, GLN241, GLN260 and GLN319 on his father's line (Fig. 1a).

- The siblings GLN220, GLN241, GLN260 and GLN319 share a 2nd-degree relationship with GLN237A (Supplementary table 8). Their relationships with GLN270B, who is very low coverage, is problematic as the p_0 value from READ for each individual appears just below the threshold of the 2nd-degree relatedness. By looking at the binomial-PMR approach, we can confirm that they are 2nd-degree related to GLN270B with posterior probabilities 0.94, 0.98, 0.94 and 0.99 respectively (see binomial-PMR distribution plot). Hence, the siblings GLN220, GLN241, GLN260 and GLN319 must be nephews of GLN237A and grandsons of GLN270B (Fig. 1a).

- GLN285A shares a 2nd-degree relationship with GLN245B and GLN261 (Supplementary table 8). The only possibility is that he is the nephew of GLN245B and the grandson of GLN261, via his father as he does not carry the same mitochondrial haplotype as his uncle (Fig. 1a).

- The siblings GLN256 and GLN261 are 2nd-degree related to GLN237A and GLN270B (Supplementary table 8). They can only be the nephews of GLN237A and the grandsons of GLN270B (Fig. 1a).

Pedigree B

- The siblings GLN301 and GLN306 share a 2nd-degree relationship with GLN280, GLN291, GLN300, GLN298 and GLN305 (Supplementary table 8). They can only be the nieces of GLN280, GLN291 and GLN300 and the granddaughters of GLN298 and GLN305 via the father's line, because of the non-matching mitochondrial haplotypes with the grandmother GLN298 and the uncles GLN280, GLN291, GLN300 (Fig. 1a).

- GLN309 is 2nd-degree related to GLN301 and just below the threshold of the 2nd-degree relatedness with GLN306 according to READ (Supplementary table 8). By looking at the binomial-PMR distribution, they are 2nd-degree related with a posterior probability of ~1 (see binomial-PMR distribution plot). GLN309 must be the nephew of GLN301 and GLN306, via his father as he does not share the same mitochondrial haplogroup with his aunts (Fig. 1a).

- In the pair mother-daughter GLN288 and GLN289B, for which we were not able to determine who was the mother and the daughter as they are both adults, the individual GLN288 shares a 2nd-degree relationship with the individual GLN291. The READ results show a p_0 value just below the threshold (Supplementary table 8) but the binomial-PMR distribution confirms this relationship, indicating a posterior probability of 0.83 (see binomial-PMR distribution plot). This 2nd-degree relationship cannot represent an ascendent link through the top of the tree, as GLN288 would share other relationships with the relatives of GLN291, therefore it must be a descendant link. She cannot be the niece or half-sibling of GLN291 because she would also be 2nd-degree related to his siblings and/or parents. She must be his granddaughter. The individual

GLN289B does not share a 2nd-degree with GLN291, so she must be the daughter of GLN288, and not her mother (Fig. 1a).

Unlinked individuals

- The remaining related pairs GLN299 and GLN302, GLN287 and GLN310B, and GLN211A and GLN211B do not share any 2nd-degree relationship with the other individuals from the group (Fig. 1a).

- Among the individuals categorized as "Unlinked unrelated individuals", the adult females GLN207B, GLN232B, GLN242, GLN243B, GLN246, GLN284 and GLN294, the subadult females GLN313 and GLN326, and the subadult males GLN229 and GLN308 do not share any 2nd-degree relationships with the rest of the individuals buried in the necropolis (Fig. 1a).

- However, the only adult male GLN311 categorized as "Unlinked unrelated individuals" does share a 2nd-degree relationship with GLN270B, but none with his brother GLN231A, his son GLN237A, and neither with the 2nd-degree related individual GLN320. GLN311 carries the Y-chromosome haplogroup H2m, different from the main lineage haplogroup G2a1a. Given that both individuals share neither the same mitochondrial haplogroup nor the same Y chromosome haplogroup, an alternative possibility is that GLN311 is a grandson on the maternal side. However, this scenario seems problematic as GLN311 is not 2nd-degree related with GLN237A, who would be his uncle in that case. Given the uncertainty, we chose not to plot this connection in the main pedigrees (Fig. 2).

Exploration of patrilocality using READ data

To explore the patrilocal pattern with an analytical approach, we used p_0 values given by READ and calculated the average of every pair for each individual with every individual from the rest of the group, as performed in Villalba-Mouco et al. 2021[46]. The mean p_0 value represents the average degree of relatedness of each individual to the whole group: the lower this value is, the more related the individual to the group, overall. We calculated this value separately for adults and subadults and plotted this value for each individual in both Extended Data Fig. 7a and 7b, using the following R packages: ggplot2 (v3.3.2)[47], tidyverse (v1.3.2)[48], magrittr (v1.5)[49], data.table (v1.13.0)[50], janitor (v2.0.1)[51]. We performed a Wilcoxon test to assess if the genetic sex and age groups are significantly differently related. The test is significant for the whole group (p-value = 5.156e-06) and for the adults (p-value = 1.375e-05) but not for the subadults (p-value = 0.1067). Note that these p-values cannot be directly compared due to the varying samples sizes in each case.

Supplementary Note 3. Imputation and IBD sharing analysis
Harald Ringbauer, Ainash Childebayeva, Maïté Rivollat

In order to double-check the reconstructed pedigrees, we imputed higher-coverage sequence data, and then ran Identity-by-descent (IBD) sharing analysis on imputed data.

Imputing the ancient DNA data

Samples were imputed using the software GLIMPSE[52] using a default pipeline developed by the authors of the software (https://odelaneau.github.io/GLIMPSE/tutorial_b38.html). We set the threshold for imputation with GLIMPSE at 500,000 SNPs covered on the 1240k panel (n=72)[53]. First, .bam files with 2 base pairs trimmed from each end of every read to account for aDNA degradation were processed with bcftools to determine genotype likelihoods using the 1000G panel[54] as a reference. Imputation was then run with GLIMPSE_impute on genomic chunks of 2,000,000 base pairs with the buffer size of 200,000 base pairs. The chunks were then ligated using GLIMPSE_ligate, and fully phased haplotypes were determined using GLIMPSE_sample.

Inferring IBD sharing

The output of the imputation of GLIMPSE was analyzed using the software ancIBD[55] (version 0.2a, https://pypi.org/project/ancIBD/). Using he recommended settings of the Python software package (v 2.7.18), we inferred IBD segments at least 8 cM long between all Gurgy individuals with >500,000 of the 1240k SNPs covered at least once (n=72). For each pair of individuals with at least one detected block of IBD (n=2412 pairs), we recorded summary statistics of the IBD sharing and report both number and total length of IBD>8, 12, 16 and 20 cM, as well as longest IBD block (Supplementary table 10).

Comparing IBD sharing with inferred pedigrees

To evaluate the concordance of IBD sharing and the inferred pedigree, we created an automatic tree crawler that reports the degree of relatedness for a given pair of individuals (implementation available at https://github.com/hringbauer/ibd_gurgy/blob/main/notebooks/tree/read_tree.ipynb). The program takes as input a table of all individuals with both their parents and constructs a directed graph, where each individual points towards its two parent individuals. The software then runs a modified Breadth-first search algorithm. First, for individual one at each ancestral node (corresponding to one individual) the degree of separation from the target sample is stored. Running the Breadth-first algorithm from the second individual, we then identify the first common ancestor. If no common ancestor exists, the algorithm returns relatedness 0. Combining the distance of individual 1 and individual 2 to the common ancestor, we obtain the degree of relatedness. Additionally, we store whether one individual is directly ancestral to the other (equivalent to the distance of one individual to the common ancestor being 0). Moreover, we store half-sibling relationships: The algorithm checks when the first common ancestor is found, and also if this ancestor's partner is a common ancestor of the same depth.

We then compared the inferred pedigree relatedness (reconstructed without IBD) with inferred IBD sharing between all 2556 pairs of 72 individuals with sufficient coverage. The results are given in Supplementary table 10 and plotted in the Extended Data Fig. 1b and at https://doi.org/10.5281/zenodo.7224898. In particular, we looked at IBD sharing for all pairs

of a given degree of relatedness (1st-8th degree). We further grouped relatives as being ancestral to each other or being related via two shared parents (i.e., via full siblings), as IBD sharing is expected to be different because the number of meioses differ.

Considering the 1st-, 2nd- and 3rd-degree related pairs, the IBD sharing analysis matches perfectly with previous reconstructed biological relatedness found via the output of READ and lcMLkin (Supplementary Note 2), confirming both the reliability of IBD sharing method as well as the robustness of our reconstructed pedigrees. The IBD analysis also confirms the parent-offspring relationship between GLN285A and GLN285B, which *lcMLkin* determined as siblings, but which was clearly inconsistent with both the tree reconstruction and the PMR-window analysis (Supplementary Note 2, Extended data Fig. 1a and 1b).

We note that beyond the 3rd degree, overlaps between IBD clusters start to appear, and it is no longer possible to assign a single degree relative IBD cluster anymore (Extended Data Fig. 1b). This is expected given the biological variation of IBD sharing (and consequently all genetic relatedness). However, we can still detect definite recent genealogical links as multiple long IBD are distinctly shared for most individuals up to six degrees apart.

Pedigree A

Within Pedigree A, one pair yielded a strong inconsistency between its relatedness in the pedigree and the IBD sharing results: GLN202 and GLN243A, represented by the light blue dot on the upper end of the 3rd-degree cluster on Extended Data Fig. 1b (Supplementary table 10). This had been identified already via READ and the binomial-PMR method where they appear 2nd-degree related (see binomial-PMR distribution plot), while they are only 4th-degree related along the paternal line from the pedigree. IBD analysis further shows that GLN202's mother, GLN315, shares a 3rd-degree relationship with GLN243A. The only explanation would be an extra connection via GLN243A's unsampled mother, such as GLN315 being the niece of GLN243A's mother. However, GLN202 and GLN268, brother of GLN243A, were found to be unrelated via READ. This might be explained by the poor coverage of GLN268. If we look at further relatedness, GLN202 and his son GLN201 show some more connections with individuals related in a descendant line with GLN243A: his daughter GLN262, his grandchildren GLN214 and GLN236B, and his nephew GLN268A (see IBD plots), indicating extra-connections between these two branches that we are not able to identify given the missing individuals in our data.

If we inspect each cluster separately for every degree of relatedness, determined according to the pedigrees (https://doi.org/10.5281/zenodo.7224898), we detect several inconsistencies in the clusters of expected 7th and 8th degrees of relatedness, where the pairs are more closely related than expected according to the pedigrees. These pairs represent two different groups of individuals (Supplementary table 10).

- The pair GLN232C and GLN285A should be related at the 7th-degree but are clearly not within the cluster. Given their distant position in the Pedigree A along the paternal line, the only way to explain a closer connection would be along their maternal line, which is unverifiable as both their mothers are missing (see IBD plots at https://doi.org/10.5281/zenodo.7224898).

- The other pairs of individuals found outside of the clusters of 7th and 8th degrees relationships are all connected to each other: GLN206 and GLN208 in one hand, GLN256, GLN261, GLN250, GLN245B on the other hand. Both groups are more related to each other than expected from the reconstructed pedigree. Given the overall consistency of the pedigree, we propose once again a connection via the maternal line, more likely via the missing mother of GLN256 and GLN261, or a missing sister related to these individuals, as the pair GLN206 and GLN208 are consistently more closely related to the siblings GLN256 and GLN261 than to the cousins GLN250 and GLN245B (see IBD plots at https://doi.org/10.5281/zenodo.7224898).

Pedigree B

All links established in Pedigree B are consistent with the inferred IBD sharing. Both links between the mother-daughter pair GLN288 and GLN289B with GLN291 are confirmed. Moreover, the direct line between GLN291 and GLN288 is further evidenced by the IBD analysis (Supplementary table 10). This is confirmed by a 3rd/4th degree shared between GLN288 and both GLN298, mother of GLN291, and GLN280, brother of GLN291, who are respectively her great-grandmother and grand-uncle. The coverage of GLN289B does not allow for this individual to be included in the IBD sharing analysis.

Additional connections

The IBD sharing analysis also allowed us to identify extra-connections with previously unlinked individuals that we could not detect with the other methods READ and lcMLkin, which are limited in their resolution to identifying 1st and 2nd degrees.

- The most interesting link revealed by the IBD sharing analysis is a 3rd or 4th degree in indirect line between GLN298, mother of Pedigree B, with GLN263, an adult male of the fourth generation in Pedigree A. By looking at the individuals linked to both of these individuals, we can confirm this link with a 4th/5th degree between GLN298 and GLN321, the brother of GLN263, as well as between GLN263 and both GLN291 GLN280, the sons of GLN298. The exact relationship is not trivial to assess, especially as we do not know exactly how many degrees connect both individuals, but this IBD signal definitely links the two main pedigrees through a genealogical connection within a few generations (Supplementary table 10, Fig. 2).

- The siblings GLN211A and GLN211B, previously unlinked to the two main pedigrees, show a 4th/5th degree relatedness with the siblings GLN325, GLN265, GLN221B and GLN266. However, given the genetic distance between the individuals, and both the existence of missing individuals, and the low coverage for some present individuals, it remains impossible to establish the exact relationship (Supplementary table 10, Fig. 2).

- For two of the adult male individuals, assessed as unlinked to the main pedigrees, GLN320 was found to be as 2nd-degree related to GLN231A and GLN270B according to READ. We cannot double-check these connections with the IBD sharing analysis given the low coverage of GLN231A and GLN270B. However, results of the IBD analysis indicate at least a 4th/5th-degree connection of GLN320 with the siblings GLN256 and GLN261, as well as with the siblings GLN325, GLN265 and GLN221B. This all appears to be consistent with the previous hypothesis (Supplementary Note 2)

that GLN320 could be the grandfather, uncle, or nephew, all through the paternal line, of GLN231A and GLN270B (Fig. 1, Supplementary table 10).

- IBD sharing analysis confirmed the absence of biological relatedness between the two unrelated subadult males GLN229 and GLN308 and any other individuals buried in the site (Supplementary table 10). We can exclude up to 6th-degree relatedness, as those would most likely have at least multiple long IBD segments.

- Similarly, for the adult females unlinked to the pedigree, IBD sharing analysis confirmed very distant or non-existing links between them or with any individual from the group (Supplementary table 10, Extended Data Fig. 4).

- The individual GLN326, a 2-5-year-old girl, shows numerous connections with individuals from Pedigree A, in different parts of the tree, making it difficult to disentangle. She shares some $3^{rd}/4^{th}/5^{th}$-degree connections with the siblings GLN220, GLN241, GLN260 and GLN319, with the siblings GLN325, GLN265, GLN221B and GLN266 with whom she shares the same mitochondrial haplotype, with the nuclear family of the external paternal line of GLN275, as well as with GLN315, GLN202 and GLN201 (Supplementary table 10). On the basis of the available data, it not possible to establish the exact position for this individual, as multiple relationships are plausible.

Supplementary Note 4. Runs of Homozygosity
Harald Ringbauer

We inferred runs of homozygosity (ROH) using the software hapROH (v0.60)[56]. Applying default parameter settings and using the default 1000 Genome haplotypes as a reference panel, we screened all individuals with more than 300,000 SNPs covered on the 1240k capture assay (n=86). For each individual, we report summary statistics for ROH: both the number and total sum of ROH longer than 4, 8, 12, and 20 cM, as well as the maximum ROH length (Supplementary table 11, Extended Data Fig. 9c).

<u>Consanguinity</u>

None of the individuals have long ROH blocks with a total length of 50 cM or more (>20 cM), which was the threshold to identify plausible offspring of first cousins[56]. The individual GLN282 stands out, as it has the most ROH and is the only individual with two ROH longer than 20 cM (Supplementary table 11, Extended Data Fig. 9c). However, both are 20-22 cM long, indicating that this individual is more plausibly the offspring of 2nd or 3rd cousins. We conclude that there is an overall absence of close-kin unions (defined as first cousins or more closely related) in the sample, as not a single individual yielded ROH typical of such unions.

<u>Estimating population size from inferred ROH segments</u>

We estimated effective population size (denoted N_e) of the Gurgy sample based on the inferred ROH, which can be interpreted as the effective size of the recent ancestry pool. For this analysis we used the function “MLE_ROH_Ne” of hapROH, and ran it with the recommended settings. Using all segments of length 4-20 cM, we arrived at an overall estimate of N_e=1834.6 (95% CI 1631-2077; Table Supplementary Note 26).

Inferred N_e	CI 95% lower	CI 95% upper	Sample Size	ROH Length Analyzed [cM]
1747.9	1532.7	2007.1	86	4-8
2049.2	1480.6	2956.2	86	8-12
2592.3	1575.7	4698.3	86	12-20
1170.4	650.2	2435.9	86	20-30
1834.6	1631.1	2077.7	86	4-20

Table Supplementary Note 26. Effective population size estimates based on the inferred ROH.

We note that N_e estimates are typically lower than census size estimates of the ancestral populations (by a factor of 3-10-fold, due to varying offspring distributions and multiple generations living simultaneously), and that they effectively measure the pool of ancestors at various time depths (according to the ROH length)[57].

The presence of ROH longer than 4cM in 82 out of 86 individuals with sufficient coverage for this analysis, with 41 of the individuals carrying even ROH longer than 8cM (Extended Data Fig. 9c) suggest that most pairs of parents were related to each other via co-ancestors within the preceding 5-30 generations[56]. This finding further supports the scenario where a group consistently brought in partners from a limited number of allies and/or a select few groups, coherent with the observed pattern of controlled female exogamy. For

comparison, in many Early Neolithic populations from Central Europe most individuals have no ROH longer than 4cM[58], which indicates larger pools of ancestors, or a pioneer phase sourced from long-distance demes, while individuals from the western part of the Mediterranean wave of Neolithic diffusion show a higher background relatedness[59].

Supplementary Note 5. Within-group diversity
Maïté Rivollat

f-statistics, IBD sharing and heatmaps

Outgroup f_3-statistics were calculated using qp3Pop from ADMIXTOOLS[60]. To investigate the group diversity, we calculated outgroup f_3-statistics of the form f_3(individual, individual; Mbuti.DG), obtaining a value for each pair of individuals (Supplementary table 12).

Following the method described in Supplementary Note 3, we calculated the shared IBD for all the pairs formed by the 72 individuals with a coverage >500,000 SNPs (Supplementary table 10).

Using both sets of values, we created two similarity matrices which were then used to generate heatmaps using the *heatmap.2* function of the R-package gplots (v3.0.4)[61] and *dyplr* (v1.0.9)[48]. In order to investigate the general diversity within the group, we constructed both heatmaps based of f_3-statistics (n=94) and IBD statistics (n=72) (Extended Data Fig. 4a and 4c).

To explore the diversity among adult females more specifically, we restricted the analysis to the adult females who were considered to be exogenous. We also built both types of heatmaps for females who are not descendants of the main lineage (therefore excluding GLN325, GLN212 and GLN213), and excluding all subadult females, as they all have parents within the pedigrees. However, we decided to include GLN288 as she is distantly related to the Pedigree B. 16 adult females were used for the f_3-statistics distance-based heatmap (Extended Data Fig. 4b) and 12 for the IBD-based heatmap (Extended Data Fig. 4d).

Results

The heatmap constructed from the outgroup-f_3-statistics mirrors the relatedness patterns (Extended Data Fig. 4a). Both main pedigrees are clearly visible, forming two distinct clusters, in which each nuclear family forms its own sub-cluster. However, f_3-statistics do not have enough resolution to detect links between both pedigrees. The additional small groups of related individuals are also clearly visible along the diagonal of the matrix. Some extra links also appear between the different clusters, but f_3-statistics do not allow further interpretation on these connections. Note also that the uncertainty in the estimates of the outgroup-f_3-statistics is presented here, and could explain potential random links.

The IBD-based heatmap, although constructed using less individuals, by the nature of the analysis itself (Supplementary Note 3), is able to detect deeper relationships, allowing for the identification of more distant connections and the visualization of the interconnections between the different nuclear families that have been detected by the IBD sharing analysis (Extended Data Fig. 4c).

When restricting the analysis to include only the exogenous adult females, the scarcity of biological connections is striking. All of the adult females are either unrelated, or only very distantly related, to each other (Extended Data Fig. 4b and 4d), except for three pairs of individuals.

As expected, GLN298 and GLN288, visible in both f_3-statistics and IBD heatmaps, have already been assigned as 3rd-degree related, and have been discussed above (Supplementary Note 3). The two other pairs, GLN315 and GLN242, and GLN232B and GLN294, are visible only in the f_3-statistics heatmap. Unfortunately, three out of these four

individuals did not meet the coverage threshold allowing them to be included in the IBD sharing analysis. However, f_3-statistics yield similar values for GLN315 and GLN242, and GLN232B and GLN294 when compared to GLN298 and GLN288. Assuming that these f_3-values are roughly corresponding to a 3rd/4th degree between two individuals, we propose that these two pairs are connected to each other, somewhere around the 3rd/4th degree (Fig. 2). Following the same rationale, the f_3-values also show that the adult female GLN232B shares a link with the unlinked subadult female GLN326, likely around the 3rd-degree, as well as with the adult male GLN302, likely around the 3rd/4th degree (Fig. 2). Still using the f_3-values, the second unlinked subadult female GLN313 shows a 3rd or 4th-degree connection with the siblings GLN287 and GLN 310B. We do not claim to perfectly assess a strict relationship here, but given the extremely rare connections between the adult females, we felt that these were worth mentioning.

Supplementary Note 6. Mitochondrial haplogroup analysis
Maïté Rivollat, Fanny Mendisco, Wolfgang Haak

Methods

We generated complete mitogenomes using an in-house mitochondrial capture probe set following the method published in Maricic et al. 2010[62] and modified according to Haak et al. 2015[63]. We mapped reads from mito-capture data using samtools (v1.3.1)[64] (mapping quality ≥30) to the revised Cambridge reference sequence[65], using the circular mapper implemented in the EAGER pipeline[66]. We called consensus sequences using Geneious R8.1.974[67] and used HaploGrep 2 (v2.4.0) to determine mitochondrial haplogroups[68] (Supplementary table 5).

Results

The complete mitochondrial genomes of 78 individuals are reported here (Supplementary table 5). The complete mitogenomes of 22 individuals were previously published in Rivollat et al. 2020[39], and partial genomes (HVRI and a set of 18 SNPs) of 35 more individuals in Rivollat et al. 2015[37], that are reanalysed in this study (Supplementary table 5). Out of these 35 individuals, six haplogroups could be refined and reassigned based on complete mitochondrial genome data. GLN249 haplogroup changes from U5 to K2b1a, GLN285B from V to K1a+195, GLN322 from H1 to K1a2, GLN264 from H1 to J1c1b1, GLN292 from H1 to H4a1a+195, and GLN311 from U5 to U1a1a.

Overall, the quality rank given by HaploGrep 2 is above 95% (Supplementary table 5). For two individuals, GLN279 and GLN270B, the quality rank is below 90%. GLN279 carries the haplogroup J1c1 (76%) but has many missing polymorphisms. However, as this individual is the sister of GLN207A who also carries the haplogroup J1c1, we can confirm this haplogroup for GLN279. GLN270B has too many missing polymorphisms to characterise their haplogroup, and remains very similar to the revised Cambridge reference sequence[65]. Therefore, we did not call any haplogroup for this individual.

In total, 36 different mitochondrial haplogroups have been found among the Gurgy individuals (Supplementary table 5). This high diversity is expected given the strong patrilocal system practiced in the group. At each generation, new females come from another group and bring along new mitochondrial haplogroups. Those are not transmitted further than to the next generation, when the new female offspring leaves the group. According to such a system, a high mitochondrial diversity is maintained, while the Y chromosome diversity is strongly restricted, as observed in numerous patrilocal populations[69].

Several of the assigned haplogroups are not reported in their exact form amongst modern populations in Phylotree[70] (https://www.phylotree.org), though the mitogenomes are complete and well covered. For instance, the four siblings GLN260, GLN220, GLN241 and GLN319 carry the haplogroup N1a1a1a, with a quality rank of 93.15% (Supplementary table 5). All four share the same haplotype, and the sequence haplotype can be determined precisely. However, since this haplogroup was common in Neolithic populations but very rare today, the derived branches of N1a1a are not fully resolved in phylotree and thus result in lower quality ranks. We made similar observation for K1a3*1, shared by individuals GLN211A and 211B.

Mitochondrial haplogroups for 99 individuals

The mitochondrial haplogroup of each of the 94 individuals with nuclear data was used to reconstruct and confirm the pedigrees. With the exception of GRG102/GLN270B, for who we could not assign the mitochondrial haplogroup (see above), each haplogroup call agrees with the position of the individual in the pedigrees (Extended Data Fig. 3b).

The mitochondrial haplogroup of six additional individuals was assigned: two complete mitogenomes for GLN219 (H1) and GLN292 (H4a1a+195), and partial mitogenomes for GLN264 (J1c1b1), GLN295 (U5b1d1), GLN314 (J), and GLN312 (H26) (Extended Data Fig. 3b).

Haplogroup U5b1d1 (GLN295) and J (GLN314), in the form that we recovered, are unique amongst the individuals buried at Gurgy.

Haplogroup H1 is the most common sub-haplogroup of haplogroup H and is shared by many individuals (n=13). It is therefore not possible to discern whether the haplogroup sharing with individual GLN219 was specific or random chance.

Haplogroup H26, carried by GLN312, is also carried by GLN313, but with different private mutations (Supplementary table 5). With GLN312 being poorly covered, this partial mitogenome does not allow for a confident haplotype call.

Haplogroup H4a1a+195 in GLN292 is also carried by the sisters GLN306 and GLN301 of Pedigree B, and the subadult GLN235 of Pedigree A. A connection might exist between these individuals through their maternal line, and the spatial proximity in the north-east part of the site where the individuals from pedigree B are grouped also suggests a link between GLN292 with the two sisters GLN306 and GLN301.

Finally, haplogroup J1c1b1, carried by GLN264, is carried by two other individuals, the sisters GLN277 and GLN307 from Pedigree B. The rarity of the haplogroup in the group suggests a link between these three individuals, although the position of the grave of GLN264 is not in close proximity with the two sisters.

Gurgy mitochondrial diversity

Given the extremely high number of related individuals in the group, the calculation of the mitochondrial diversity is not straightforward. Therefore, a comparison with published whole-population data, which assumes a random sample of unrelated individuals, would only be possible from exogenous females and additional lineages from unrelated individuals. Thus, if we take the number of different mitochondrial DNA (mtDNA) lineages among unrelated exogenous females and additional unrelated individuals, we observed 35 unique mitochondrial haplotypes among 57 such individuals, resulting in a proportion of 0.614. Applying the same principle to the data from Hazleton North[71], which presents the only suitable parallel to Gurgy at the moment, results in a proportion of 0.618. In comparison, the Early Neolithic LBK cemetery Derenburg[58] which has been used for a longer period of time and for which only very few biological relationships have been described returns a proportion of 0.75. A random, chronologically dispersed, cross-regional sample of the French Neolithic meta-population[39] results in a proportion of 0.84. We are not necessarily expecting to reach such a high proportion within the perimeter of the mating network of Gurgy, but we note that the mtDNA diversity is not drastically reduced, and that we observe new mtDNA haplotypes in each generation, within in each lineage.

Supplementary Note 7. Y chromosome haplogroup analysis

Maïté Rivollat, Adam Benjamin Rohrlach

Methods

A tiled capture assay of sites on the Y chromosome, called YMCA[72], was applied to all genetically determined males (n=57). We assigned Y-chromosome haplogroups following the method described in Rohrlach et al 2021[72], according to the ISOGG SNP index v.15.73, last downloaded 11/07/2020 (Supplementary table 7).

Results

57 Y-chromosome haplogroups are reported here, amongst which 13 were previously published in Rivollat et al. 2020[39], and 6 in Rohrlach et al 2021[72] (Supplementary table 7).

Only two Y haplogroups from different major subclades are present at Gurgy, G2a2b2a1a2 (n=51) and H2m (n=6). Given the strong patrilocal signal and the common ancestor to almost all the individuals in the site, this pattern is not surprising. Some of the individuals carrying the G2a haplogroup are not well covered, and so the resolution of their terminal branch is limited. However, as they belong to the same male lineage, they must carry the same set of mutations. The haplogroup H2m is the only external lineage brought in the group by the union of a daughter (GLN325) of the main lineage.

Phylogeography of the Y-chromosome lineages

Both derived haplogroups G2a2b2a1a2 (G-L1266) and H2 (H-P96) (specifically H2m here as defined in Rohrlach et al 2021[65]) are characteristic for incoming farming groups[39,53,72-74]. G2a2b is commonly found among male LBK individuals from Eastern Germany and Central Europe[39,53,58,73-75], but not elsewhere in France or in Europe during the Neolithic. This specific haplogroup is, however, only present in Gurgy. Contrastingly, H2m is exclusively found in southern European sites and, using this marker, allowed researchers to track the Neolithic route of migration of humans along the Mediterranean coast, and then northward to France and eventually to Ireland[22,72,76]. For example, H2m is found in the Cerny site Fleury-sur-Orne, in Normandy, in the same cultural area[22]. Gurgy is a clear example of the arrival of both routes of Neolithic migration in western Europe, where genetic signals from both streams meet.

Surprisingly, no Y-chromosome haplogroup inherited from the hunter-gatherers has been found in Gurgy, such as I2a1a or I2a1b, while they are predominant in other regions, like southern France or the British and Irish Isles[39,77,78].

Supplementary Note 8. Human Leukocyte Antigens (HLA)
Rodrigo Barquera, András Szolek

Method

Using an in-solution capture strategy based on modified immortalized probe sequences[79], target immunity genes sequences were enriched via in-solution capture[80,81]. After enrichment, captured library pools were single or paired-end sequenced (Supplementary table 1) on the Illumina Hiseq 4000 (Illumina, Inc.) platform, providing on average 20 million reads. We applied a development version of OptiType (v1.3.2)[82] to sequence data from FASTQ files merged from all available libraries, mapped against OptiType's custom Human Leukocyte Antigen (HLA) reference panel containing 1025 alleles with "common" or "intermediate" CIWD 3.0 designation (https://github.com/FRED-2/OptiType, tag GRG) using reads no shorter than 40 bp. We obtained allele calls for the HLA class I (*HLA-A*, *HLA-B*, *HLA-C*) and class II (*HLA-DPA1*, *HLA-DPB1*, *HLA-DQA1*, *HLA-DQB1*, *HLA-DRB1*, *HLA-DRB3*/*4*/5) genes.

For the HLA analyses, only individuals where both chromosomes yielded unambiguous typings, or ambiguous ones that could be resolved beyond any reasonable doubt, were kept. Ninety-three out of the total 1458 raw allele calls (6.3%) were overruled in favor of runner-up alleles based on anomalous coverage patterns induced by reads mapping to alleles of multiple loci, and/or consistency with related, higher quality samples. Haplotypes were assigned based on the genetic genealogy of the analysed individuals, backed by previously reported frequencies and properties such as linkage disequilibrium (LD)[83-85]. Given the fact that the HLA-DP region is far away enough not to allow LD between these genes and the rest of the HLA class II genes, *HLA-DPA1* and *HLA-DPB1* should not be considered as part of the extended HLA haplotype, and its inclusion as part of the haplotype follows mendelian inheritance patterns found in the genealogy only. To add to the readability of the HLA data on the genealogy (Fig. 1), we reduced the whole haplotype nomenclature to a four-digit combination signaling the *HLA-A*, *HLA-B*, *HLA-C* and *HLA-DRB1* alleles present in the haplotype (Supplementary table 14, Extended Data Fig. 5a). Hence, haplotype HLA-A*02:01~B*27:05~C*02:02~DRB1*04:01~DRB4*01:03~DQA1*03:01 ~DQB1*03:01~DPA1*01:03~DPB1*04:02 would be transformed into A.02.27.02.04. A complete list of all haplotypes and their equivalence for the genealogy can be found in Supplementary table 14.

Results

A total of 63 different HLA haplotypes (from 164 total haplotypes) could be detected, all of which yielded alleles previously reported for similar contexts (Supplementary table 14). Given the fact that the Gurgy individuals are almost all genetically related to a certain extent, it is not possible to accurately calculate allelic frequencies. Instead, we present the genetic genealogy with the haplotypes inherited through seven generations. Common alleles include those previously reported for Neolithic and Bronze Age Eurasian populations[58,86,87], including HLA-A*02:01, HLA-A*24:02, HLA-A*31:01, HLA-B*15:01, HLA-B*27:05, HLA-DRB1*08:02 and HLA-DRB1*11:01. Apart from being able to report non-statistically inferred HLA haplotypes for Neolithic Europe, we were also able to detect two recombination events in the genealogy, one among HLA class I genes and another in the HLA class II region (Extended Data Fig. 5a). The first of these events involves individual GLN267, who carries a new maternal haplotype, arising from the recombination of the *HLA-A* allele from haplotype A24.51.05.13 (A*24:02) and haplotype A31.15.04.11, resulting in

the new haplotype A24.15.04.11. The presence of haplotype A31.15.04.11 in two siblings (GLN227 and GLN268A) confirms the recombination event. The HLA class II region recombination event was detected in individual GLN245B. Haplotype A02.27.07.04 in individual GLN261 underwent a recombination event in the whole HLA class II haplotype (HLA-DRB1*08:01~NULL~DQA1*04:01~DQB1*04:02 X HLA-DRB1*04:01~DRB4*01:03~DQA1*03:01~DQB1*03:01) to form haplotype A02.27.07.08 in individual GLN245B. The newly formed haplotype is confirmed by its presence in individual GLN245A, and by the presence of the original A02.27.07.04 haplotype in GLN285A and his son (GLN285B).

It is of note that both HBV-infected individuals (GNL201 and GNL258) bear alleles that have been reported to be associated with HBV persistence and infection chronicity: alleles HLA-DRB1*11[88,89] and -DRB1*13[90] are present in the HLA genotype of GNL201, while HLA-DQB1*03:01[88,89,91] is present in both individuals (homozygous in GNL258). However, HLA-DRB1*13 has been shown to have also a protective effect, making the role of this allele controversial[92].

Supplementary Note 9. Analysis of variants associated with phenotypic traits
Ainash Childebayeva, Maïté Rivollat

For each of our individuals, we investigated the genotypes of 72 SNPs associated with phenotypes of interest[53,93], which includes the 41 SNPs from in the HIris-Plex-S tool for hair, eye and skin pigmentation developed on modern European data (https://hirisplex.erasmusmc.nl)[94-97]. We calculated the genotype likelihood using the UnifiedGenotyper module of the Genome Analysis Toolkit (GATK) v.3.5. These calculations were based on the number of reads from our bam files (phred-scale mapping quality score (MAPQ)>30 and base quality score (BASEQ)>30) for each position to determine the presence of the ancestral (non-effect) or derived (effect) alleles. The results from this analysis are provided in Supplementary tables 15 and 16.

Considering specifically the SNPs involved in pigmentation, we reconstructed the most-likely phenotypes using the HIris-Plex-S tool, using the weight of each SNP in the determination of the probabilities for phenotypic assignments. For eye color, following Walsh et al 2012[97], we set our probability threshold at p=0.7, corresponding to a ~95% probability of correctly assigning eye colors to our samples (n=52). We found that if we lowered the threshold to p=0.5 (~90%), we could assign an eye color for an additional 13 individuals. Following Walsh et al 2014[96], we assigned a hair color for 80 individuals. Finally, for the level of skin pigmentation, following the indications in Chaitanya et al 2018[94], we assigned levels of pigmentation to 70 individuals. Overall, the individuals from Gurgy represent the full spectrum of variation in skin, hair and eye color pigmentation, ranging from 'Blond/Dark-Blond' to 'Black' for hair colour, including ten individuals with red hair, and from 'Very pale' to 'Dark-Black' for the skin pigmentation. The eye colors were also variable, including blue (N=23) and brown (N=42). It should be kept in mind, however, that these different pigmentation spectra are based on modern European populations and may not reflect the actual appearance of prehistoric populations, especially during the Neolithic, prior to the Bronze Age migrations.

Supplementary Note 10. Population genetic analyses
Maïté Rivollat

Datasets and panels

We merged our new data with the HO panel (~600k SNPs)[60,98] with ancient data published for the ancestral populations and Mesolithic and Neolithic groups in western Eurasia. See the complete list in Rivollat et al. 2020[39]. We also merged our data with the same published ancient data to the 1240k SNP panel[53] including 300 present-day individuals from 142 populations sequenced to high coverage[99] and used this dataset, restricted to the autosomes, for subsequent genome-wide analyses. We excluded individuals with less than 10,000 covered SNPs on the HO panel.

PCA

Using the HO panel we performed a PCA using the "smartpca" software v10210 (EIGENSOFT; Extended Data Fig. 9a)[100]. We computed principal components from 777 present-day west Eurasians. To compensate for the incomplete nature of the ancient data, published ancient individuals from Mesolithic and Early and Middle Neolithic periods (before ~4000 BCE) from western Eurasia were projected using the options lsqproject: YES, and shrinkmode: YES.

In the PCA, the Gurgy individuals form a homogenous genetic cluster (Extended Data Fig. 9a). Although the Gurgy individuals form a tight cluster, the main ancestor of Pedigree A (individual GLN270B) is slightly shifted upwards on PC2, but this variation is likely explained by the fact that the coverage for this individual was much lower (Supplementary table 1).

qpAdm

We applied qpAdm to the 94 individuals available for Gurgy to estimate proportions of Anatolian Neolithic and Loschbour ancestries (ADMIXTOOLS)[63]. We chose Anatolian Neolithic individuals as the main distal source of Neolithic ancestry from Anatolia[74,101] and the Mesolithic individual from Loschbour as a representative of the hunter-gatherers present in the region[39,102]. We used a set of ten outgroups: Mbuti.DG Papuan.DG Onge.DG Han.DG Karitiana.DG Israel_Natufian Ethiopia_4500BP.SG Ust_Ishim_HG_published.DG Russia_MA1_HG.SG Italy_Villabruna (Supplementary table 17, Extended Data Fig. 9b) and used the parameter useallsnps: YES. We observe that Gurgy individuals carried a consistent proportion of Loschbour ancestry, ranging between 7.8 – 22.2% (15.62% on average), common during the Middle Neolithic)[22,39,63,74,103,104].

Following the results from Rivollat et al. 2020[39] where Goyet Q2 ancestry was found in the Gurgy group, we explored the presence of this Magdalenian-derived ancestry at the individual level using the model proposed by Villalba-Mouco et al[103] (Supplementary table 17). Twelve individuals carried a small proportion of Goyet Q2 ancestry (from 3.6% to 9.2%). If we look at their position in the pedigrees (Supplementary table 17), a few of these individuals are isolated, but two clusters seem to show the transmission of this component from a generation to another. One transmission is visible from the father GLN237A to one son GLN265 and to two grandsons GLN216 and GLN276, although it cannot be detected in any of their parents. This raises questions about the detection of that component with the qpAdm method. One explanation would be that the proportion of Goyet Q2 component is too

low in both parents to be detected in our model, but, when combined in both sons, it becomes important enough to be quantified. It could also be explained by the specific SNPs present or absent in each individual. The other transmission of Goyet Q2 ancestry is visible from the man GLN261 and his brother GLN256, as well as his son GLN245B and one of his grand-sons GLN258, whose mother GLN249 also carries Goyet Q2 component. It is worth noting that the son GLN258 carries more Goyet Q2 component (8.6 ± 2.7%) than both parents (GLN245B = 4.6 ± 2.6% and GLN249 = 6.2 ± 2.6%).

DATES

We used the method DATES v.753[105] to leverage patterns of ancestry covariance to estimate the date of admixture between Anatolia_Neolithic and Loschbour (Supplementary table 18).

We applied DATES to the entire pooled group as well as to each individual separately (Supplementary table 18). Here, we assumed an average generation time of 28 years[106]. The individual estimates are overall consistent with the group estimate (34.18 ± 1.99 generations), and the old admixture date estimate between farmers and hunter-gatherers of 34.15 ± 1.84 generations before (or about a thousand years) explains the genetic homogeneity of the group.

However, a few aberrant values are observed, and are either negative or excessively large. Some aberrations are clearly due to low-coverage samples (GLN268B, GLN270B, GLN289B, GLN311). The observed threshold is about 100,000 SNPs called on the autosomes for the SNPs on the 1240k capture assay: below this limit, DATES estimates do not seem reliable, giving values of thousands of generations. Two individuals also show aberrant values even though they are well-covered and yield an important part of hunter-gatherer component (GLN220 and GLN232B).

Lastly, two individuals yield very recent admixture dates (GLN287, 3.39 ± 5.59 generations and GLN310B, 4.98 ± 3.55 generations). They are the only ones that indicate a very recent admixture event. Interestingly, these individuals are brothers, and they are not connected to the main pedigrees, and have no close link to any other individual in the group. Therefore, it cannot be ruled out that their direct ancestors truly underwent a very recent admixture event, even though the proportion of Loschbour ancestry does not differ from the other Gurgy individuals (GLN287 = 14.6% and GLN310B = 14.2%). For comparison, the individual Oase 1 has between 1.6 and 6.3% of Neandertal ancestry, calculated with the length of the fragments, giving an estimation of a Neanderthal ancestor as a 4th-, 5th- or 6th-degree relative[107]. It is also possible that this signal could occur if their parents carried similar proportions of Loschbour ancestry, but in very different regions. i.e., the parents both carry Loschbour ancestry from (approximately) the same historical admixture event, but it was non-overlapping.

Supplementary Note 11. Strontium isotope analysis on tooth enamel and inter/intra individual mobility assessment

Léonie Rey, Gwenaëlle Goude, Vincent Balter

Material, tooth growth and age estimation

Fifty-seven teeth were selected and analyzed for radiogenic strontium (Sr) isotope ratios ($^{87}Sr/^{86}Sr$), including 10 permanent first molars (M1) (for 4 females and 6 males) and 47 permanent second molars (M2) (for 19 females and 28 males), see details in Supplementary table 23. The selection was made on the basis of the availability of the teeth for this type of analysis (macroscopic preservation, age at death), previous evidence of collagen preservation[32,36], and the position of the individuals in the reconstructed pedigrees.

Most of the selected teeth were the M2 as their growth period reflects a specific moment in the early life of the individual, *i.e.,* after breastfeeding-weaning time but before periods of social changes evidenced both by funerary practices[108] and carbon and nitrogen stable isotopes[36]. When the M2 was not available, or was too worn to provide a relevant profile for analysis, the M1 was selected in order to discuss female vs. male behavior pattern in early stages of life.

The mineralization stage was determined on each tooth by using Moorrees et al (1963)[27] and Demirjian et al (1973)[109] methods as following:

Moorrees et al. 1963	Demirjian et al. 1973
Ci	A
CCO	B
Coc	
Cr1/2	C
Cr3/4	
Crc	D
Ri	
R1/4	E
R1/2	F
R3/4	
RC	G
A1/2	
AC	H

Table Supplementary Note 27. Mineralization stages*, as used in this study, according to Moorrees et al (1963)[27] and Demirjian et al (1973)[109]*

Then, age estimation of the crown formation is provided by using AlQahtani et al. (2010)[110] (Table Supplementary Note 27). According to this last reference, for the first molar (M1) the crown starts its growth on average at 0.5 years old (±4 months) and finishes its mineralization at around 3.5 years old (±0.5 years). For the second molar (M2), the crown starts its growth on average at 2.5 years (±0.5 years) and finishes its mineralization at around 8.5 years old (±0.5 years). More information about tooth development stages can be found at https://www.qmul.ac.uk/dentistry/atlas/.

All observations (anatomical variations, pathologies, stress indicators, wear, maturation stage) and external metrics were recorded and pictures of the teeth were taken. In addition, calculus samples, impressions of the crown and µCT-scans were computed whenever possible for further complementary studies[34,111].

Tooth preparation and Sr isotope measurement by laser ablation MC-ICPMS analysis

The teeth were cut into two pieces with a precision saw, along the longitudinal bucco-lingual axis, passing through the top of the mesial cusps and perpendicular to the collar (crown root junction, or crj). The mesial part was embedded in epoxy resin and polished (granulometry P4000) to obtain a smooth surface.

In tooth enamel, rasters were processed along the enamel dentine junction (edj) to cross the Retzius' striae and the transverse striations. The profiles were drawn from the apex to the crj. A laser ablation device (ESI NWR193, Omaha) was connected to a MC-ICPMS (Neptune Thermofisher, Bremen) for the measurements of Sr isotopes (LA-MC-ICPMS). The fluence of the laser was 6J/cm^2, a repetition rate of 20 Hz, a spot size of 100 µm, and a speed of 60 µm/s were used. The ablation cell was flushed with helium at 1L/min. Argon at 750 ml/min was added as an auxiliary gas for the MC-ICPMS, which was equipped with skimmer X and sample Jet cones. Blanks were measured during a period of about 5 seconds (laser off) before each measurement. We used a standard sample-bracketing method with sintered SRM1400 "Bone Ash" as the bracketing standard[112,113]. Strontium isotope measurements were corrected using the classical ^{85}Rb and ^{83}Kr corrections and the ^{87}Sr/^{86}Sr ratio of the measurement was subsequently normalized by the bracketing SRM-1400 standards with a factor depending on the position of the spot relative to the bracketing standards. Statistical analyses were performed, and graphical outputs were produced, using the *data.table*[50] and *ggplot*[47] packages, respectively, of the R software[114] (Supplementary table 23). All individual data are available at https://doi.org/10.5281/zenodo.7224898.

Geological context and determination of the local ^{87}Sr/^{86}Sr range

The funerary assemblage of Gurgy is located in the Yonne valley, on the bank of the Yonne River, in an area framed by two large distinct geological units: to the northwest, the sedimentary plains of the Paris Basin, and to the southeast, the high plateau of the Morvan massif, a small mountainous region located at the northeast end of the Massif Central. The Yonne River rises in the peat bogs of the Morvan and flows into the Seine. This position gives the microregion a complex geological context with varied ^{87}Sr/^{86}Sr compositions of soils and plants. An estimated ^{87}Sr/^{86}Sr local range is derived from values in the IRHUM database[115]: to determine the ^{87}Sr/^{86}Sr signature of a local geological unit, soil and plant samples are considered within a 100km radius from the site – no sample comes directly from the Yonne valley[115,122]. The range of ^{87}Sr/^{86}Sr values is detailed later for each regional geologic layer and shows a large variability. Plant ^{87}Sr/^{86}Sr values are preferentially used here, as they reflect a more relevant average of bioavailable Sr than soil values[116]. In a more recent study, Willmes et al.[117] mapped Sr isoscapes for the whole of France from geological units. While the range initially appears homogeneous across the Paris Basin on a geologic scale, closer observation moderates this apparent homogeneity.

The site is located primarily in the Quaternary alluvial plain, consisting of sands and gravels (^{87}Sr/^{86}Sr$_{plant}$ q3 = 0.70808 ± 0.00001, according to one sample F12-149 coming from another valley, the Marne valley, about 100km away). Immediately adjacent to this layer, moving away from the river, is the Lower Cretaceous c1 layer consisting of clays, sands, and sandstones (^{87}Sr/^{86}Sr$_{plant}$ c1 = 0.71016 ± 0.00001 according to one sample F12-147 collected in the Aube valley, also located almost 100km away)[122]. Downstream and still near the sites is the Senon plateau (Upper Cretaceous layer c2, ^{87}Sr/^{86}Sr$_{plant}$ c2 = 0.70890 ± 0.00140 according to 2 samples F11-121 and F12-146 collected more than 70 km from Gurgy) with chalk and chalky marls[122]. A few tens of kilometers further on, cretaceous layers, in the sedimentary plateau of the Paris Basin, are the pq1, e, and g layers, mainly composed of

sands, clays, and gravels, with a slightly higher $^{87}Sr/^{86}Sr$ signature ($^{87}Sr/^{86}Sr_{plant}$ pq1 and e =0.70869 ± 0.00002 and 0.71298 ± 0.00002 according to 2 samples respectively, F11-122 and F11-119)[122]. In some places, the m and q2 layers merge, on which the plant samples show a $^{87}Sr/^{86}Sr$ ratio around 0.714 (F11-117 = 0.71377 ± 0.00002 for m layer and F11-123 = 0.71432 ± 0.00002 for q2 layer)[122]. Upstream towards the Morvan massif, there is the plateau and the Jurassic formations of the Auxerrois with limestones ($^{87}Sr/^{86}Sr_{plant}$ of layers j3 to j1 from 0.70833± 0.00002to 0.71464 ± 0.00001 according to 5 samples; increasing from j3 to j1). The Morvan massif with plutonic and volcanic rocks, such as granites and gneisses, has a significantly higher $^{87}Sr/^{86}Sr$ ratio ($^{87}Sr/^{86}Sr_{plant}$ of layers 15 to 18, bk and h3 range from 0.71355 ± 0.00002 to 0.71893 ± 0.00037 according to 6 samples).

Thus, considering a 10-15 km radius around the site, which could represent the direct food supply environments, the area includes layers q3, q2, c1, c2, and j3. According to the IRHUM data compiled above, the corresponding range of bioavailable Sr isotopic ratios may be about 0.7075 to 0.7145. If only the q3 and c1 layers are considered more precisely, the local range narrows to the interval of approximately 0.7081 to 0.7102.

Quality control of the measurements

The average value of the $^{87}Sr/^{86}Sr$ ratios of the standards is 0.71325 ± 0.00062 (n=55), close to the accepted value of 0.71310[118]. The average Sr voltage, approximated by the ^{88}Sr voltage, was 7.2 ± 0.6 V (n=55) well above the ~0.5 V threshold where the ^{40}Ca-^{31}P-^{16}O polyatomic interference on mass 87 can produce unresolvable interferences[119-121].

The value of the $^{87}Sr/^{86}Sr$ ratio of the standard tends to the true value (0.71310) as the ^{88}Sr voltage increases (Extended Data Fig. 8a). The value of the $^{87}Sr/^{86}Sr$ ratio of the samples was always lower than that of the standards (Extended Data Fig. 8b) but was always higher than 0.9 V (Extended Data Fig. 8c). All the measurements in the samples produced variations of the $^{87}Sr/^{86}Sr$ ratio as a function of the estimated dental age (Extended Data Fig. 8d).

Bulk Sr isotope ratios and relative variability with respect to the local geological range

All of the human $^{87}Sr/^{86}Sr$ values range between 0.70793 and 0.71355, with an average of 0.71066, and are all included in the local geological range of 0.7075 to 0.7145. The broad variability of this bioavailable Sr local range makes difficult to understand the geological origin of the human samples studied. However, the lack of values higher than 0.714 suggests that no individual originates from the Morvan massif. The distribution of $^{87}Sr/^{86}Sr$ values follows a trend (Fig. 3) testifying to different geographical origins, without revealing any clear difference between groups of individuals.

Integration of genetic data: Sr results by families, sex, and origin status

Forty-six individuals from Pedigree A yielded Sr data, and had a mean $^{87}Sr/^{86}Sr$ value of 0.71085, ranging from 0.70801 to 0.71355 (Table Supplementary Note 28). The individuals from Pedigree B (n=4) produced $^{7}Sr/^{86}Sr$ values which ranged from 0.70899 to 0.71231 with a mean of 0.71013. The unrelated individuals (n=7) produced $^{87}Sr/^{86}Sr$ values which ranged from 0.70793 to 0.71218, and with a mean of 0.70972.

Females and males had a similar range of $^{87}Sr/^{86}Sr$ values, with similar mean values, 0.71082 and 0.71042 respectively. However, local and non-local (we define non-local as the exogenous females as identified by genetic data as well as the first generations who arrived at the site, *i.e.*, generations 1, 2 and 3) individuals had distinct $^{87}Sr/^{86}Sr$ values: non-local individuals produced lower values than locals, always less than 0.71017, with an average of

0.7094 for the females and 0.70867 for males. Local female and male individuals have similarly higher average Sr values (0.71162 and 0.71114 respectively).

	F	M
Min	0.70793	0.70813
Max	0.71355	0.71327
Mean	0.71042	0.71082
n	15	30

	F/L	F/NL	M/L	M/NL
Min	0.70902	0.70801	0.70823	0.70840
Max	0.71355	0.71017	0.71327	0.70891
Mean	0.71162	0.70940	0.71114	0.70867
n	9	6	27	3

Table Supplementary Note 28. Strontium averages for adult individuals. *F = female, M = male, L = local, NL = nonlocal. n represents the sample size.*

<u>A different picture according to generations</u>

Figure 3 shows the relationship between the $^{87}Sr/^{86}Sr$ values and the generation number, genetic sex, and residence status (local vs. non-local as defined above) for the individuals of Pedigree A, and suggests that the $^{87}Sr/^{86}Sr$ values appear to change over time. Males in the first generations had particularly low $^{87}Sr/^{86}Sr$ values, indicating that they spent their childhood in another location and arrived later in Gurgy. Moreover, each of these generations presents successively a higher $^{87}Sr/^{86}Sr$ value and non-local females for each generation present lower ratios than local individuals (males and females). To statistically test for the significance of sex, age, and generation in predicting the distance between burials, we ran a multivariate linear regression, beginning with a full interaction model including these three variables: generation, sex and age category. We used ANOVA (p-value cut-off of 0.05), and stepwise AIC (Akaike information criterion), to compare the nested models to find the simplest well-fit model. We found that the model which fits best is a model with generation and sex, as well as an interaction term between sex and generation, as predictor variables. The age group variable however could be completely removed from the model. The diagnostic plots for the models were all inspected to ensure that the assumptions of linear regression were satisfied. The coefficient of the interaction term "male:generation" was negative, meaning that per generation, the strontium ratio for females was increasing significantly faster than it was for males ($p=0.01474$).

The variation of the $^{87}Sr/^{86}Sr$ values observed through the generations does not reflect a specific mobility pathway as mentioned previously when considering the geological map. Hence, it is not possible to track the geographical origin of the individuals or generations. However, these data indicate that for each generation, the childhood of some of the individuals was spent in different places compared to the previous and following generation. It should be noted that the generations may be non-discrete and chronologically overlapping, depending on the age at which the women gave birth, and hence does not necessarily correspond to the same time periods. It seems that each generation did not settle in the parents' residential area, but in a new area, and the scale of the suggested territories is unknown. With $^{87}Sr/^{86}Sr$ values being sensitive to slight differences in the substratum, these territories could be a few hundred meters apart, or several kilometers. For the first time, these

data attest to a "short-term" exploitation of the environment that could be defined as "intergenerational territorial mobility".

The variability of $^{87}Sr/^{86}Sr$ values recorded in the enamel of females and males testifies to a different geographical origin according to the status (local vs. non-local) or biological relatedness. Moreover, in Pedigree A, a difference is recorded between the females from the local group ("endogenous females") and the females with no genetic ancestors within the local group ("exogenous females"), whichever generation is considered (Fig. 3). This strengthens the hypothesis of the external origin of "exogenous females" coming from non-local communities.

$^{87}Sr/^{86}Sr$ intra-profile variation and individual life histories

The cutting-edge methodology used here, LA-MC-ICPMS, to get high-resolution sequential $^{87}Sr/^{86}Sr$ data on enamel allows us to reconstruct a clearer picture of the lifestyle of the first farmers at Gurgy[122]. The early life mobility is clearly visible in the Gurgy individuals' childhood from the enamel $^{87}Sr/^{86}Sr$ sequences (Extended Data Fig. 8d). No recurrent individual mobility pattern can be identified according to the genetic position in the group (endogenous, exogenous, non-related), or the sex of the individual between 6 months until 8 years old, nevertheless, several individuals show clear $^{87}Sr/^{86}Sr$ variation between 4 and 6 and/or between 6 and 8 years old: GLN206, GLN221B, GLN243A, GLN250, GLN257, GLN261, GLN268B, GLN285A for endogenous males; all three exogenous males, GLN216, GLN275, GLN276; GLN325 for an endogenous female; GLN225, GLN236A, GLN248, GLN315 for exogenous females; as well as most of the individuals from the Pedigree B, and non-related individuals. The few profiles that are available for early life (first molar), i.e., between 6 months and 3 years old approximatively, also indicate $^{87}Sr/^{86}Sr$ variation during infancy, and echoes previous findings from prehistoric farmers from Mediterranean Europe[123]. However, no recurrence in the profile pattern is found, with these being variable and showing various types of movements without apparent overlapping. Some individuals show signs of mobility during the reporting period; regardless of sex, origin status or generation, the profiles indifferently increase, decrease, or vary intermittently. Several movements could be considered in terms of group mobility, whatever the group of origin, and in parallel with an exogenous genetic origin.

Supplementary Note 12. Geospatial analyses

Mélie Le Roy, Maïté Rivollat, Stéphane Rottier, Adam Benjamin Rohrlach

A geospatial analysis was performed using the ArcGIS v.10.8 software[124]. Potentially statistically significant spatial associations between each specific and combined funerary/osteological variable, and each maternal haplogroup/haplotype/family attribution and the generations were investigated.

To analyse the dispersion of the burial pits inside the necropolis of Gurgy 'les Noisats', x- and y-coordinates for each individual were defined using the centre of the burial as the origin. Global characteristics of the site were defined by the centroid and an ellipse of one standard deviation was used to compare the distributions of selected grouped data. Then, ellipses of one standard deviation for each funerary, osteological variable, and the genetic data, were measured. The orientation and size of the ellipses indicated where the studied data were distributed (at one standard deviation).

Next, spatial distance analyses were used to highlight clusters within the entire necropolis area using the software CrimeStat 3.3[125]. The nearest neighbour index was measured to identify the difference of the mean distance from the expected distance compared with the mean distance for a random distribution. This index is calculated using the ratio between the two mean distances. According to the method, the distribution can be clustered, random, or dispersed[126]. In order to identify these aggregates, we used the K Ripley's and Hotspot Analysis using Nearest Neighbour Hierarchical spatial clustering[126]. These statistics allowed us to consider only the geographical coordinates of the chosen data and were used only on osteological and archaeological data. Several clusters have been identified previously[40,127]. Among these, only three spatial clusters (subadults, adults, males) include individuals sharing a common funerary trait, e.g., same position of the body or same type of pit. All other identified clusters always exhibited only one shared trait, e.g., the same position of the upper limb, the same type of pit, etc.

Following Zvelebil and Pettitt (2013)[128] we tested the combination of archaeological data with aDNA through this innovative GIS approach.

<u>Distributions of the two families</u>

The families appeared to have an exclusive distribution through the necropolis area. However, this is not significant when considering the deviational ellipses at 2SDE (80%), even though those at 1SDE (60%) do not overlap (Extended Data Fig. 6b). The different orientation of the ellipses, added to the visual separated distribution, suggests two distinct phases. This could represent either a chronological succession of occupations, or pedigrees A and B settling the area at the same time and voluntarily keeping two defined spaces in the necropolis.

<u>Geospatial statistical analysis</u>

Given the apparent familial-relationship structure of the site, we further explored the structure of these burial relationships within each pedigree area. We applied a Mantel test[129] to check for the correlation between spatial distances (Supplementary table 20) and genetic distances using observed f_3-statistics (Supplementary table 12). The observed correlation was significantly positive ($r = 0.2190748$, $p = 0.000009$), implying that, overall, the closer two individuals are genetically related to each other, the spatially-closer they are buried.

We compared the spatial proximity to the type of relatedness (as inferred by the pedigree) that each pair shares when the pair involves an adult male and a close relative, adult

or sub-adult, male or female, offspring or nephew/niece. We fit a linear model predicting distance between burials based on two possible predictor variables: the type of relationship in the form of to/from, i.e., father/son, and the age of the younger individual (adult/subadult). We found that a square root transformation for the distance fit best via the Box-Cox transformation[130]. We then fit a mixed effect model[131] of the form sqrt(distance) ~ relationship*age, and found that the random effect of who the older individual was not required (p=0.2147). Using analysis of variance (ANOVA), we found that an additive model fit better than the full interaction model (p=0.1544), but that this model could not be simplified further, hence the final model was: sqrt(distance) ~ relationship+age (p=9.563e-05 and p=2.442e-07 for the single term models via ANOVA). The estimated coefficient for the age of the subadult was negative, indicating that subadults are buried significantly closer to their parents, than adults are to their parents.

Performing pairwise comparisons of the coefficients for the relationships[132], and adjusting the p-values using the false discovery rate (or FDR) method, we observed three groups that were significantly different (Fig 1e):

- father_daughter - father_son - the positive coefficient here indicates that sons are buried significantly closer to their fathers than daughters are (p=0.0306).
- father_son - uncle_pat_nephew - the negative coefficient here indicates that sons are buried significantly closer to their fathers than they are to their paternal uncles (p=1.67e-6).
- father_son - uncle_pat_niece - the negative coefficient here indicates that sons are buried significantly closer to their fathers than nieces are to their paternal uncles (p=4.77e-7).

This findings are in line with previous observations where preferential association between male adults and subadults under 7 years old has already been described[40]. We could not apply this analysis to the adult females as the extremely small number of observations did not allow for robust statistical inference. Therefore, we cannot rule out the possibility of a similar pattern for the adult females.

Endogenous and exogenous females

No distinct location within the necropolis can be observed between endogenous and exogenous females. In contrast, all of the adult females with offspring tend to be spatially integrated into their partner's area, and buried closely to their offspring, with the unrelated females spread across the necropolis, which may indicate non-biological connections.

Pedigree A

Within Pedigree A, no specific funerary practices were observed across the whole lineage (Supplementary table 22). No common funerary practice seems to be correlated to the age at death, nor to the sex of the individuals. However, the concentration of burials according to biological sex of the individuals is apparent in the eastern part of the necropolis, where female individuals were located/aligned on/along the outer rim of the area of the first phase of interments (generations 1 to 3).

This, then, led us to investigate potential correlations across generations or "nuclear families" (parents/offspring, siblings etc.).

Regarding the general distribution of the burials temporally along the generations of Pedigree A, we first noticed an expansion from east to west, up until the third generation. From this time, the distribution of the burial and the expansion of the overall necropolis followed a preferential orientation from north to south (Extended Data Fig. 6b).

No common funerary criteria are observed across generations, or close genetically related individuals (for example siblings or cousins) (Supplementary table 22, Extended Data

Fig. 3c). This can be affected by the fact that some of the family members died at a young age, while others grew old and started their own families, therefore acquiring a different social status within the community, which was then reflected in the funerary practices. However, no common funerary practice among nuclear families was observed either. Nevertheless, selected funerary criteria were shared by few members of some nuclear families. A nuclear family of parents (GLN245B and GLN249) and their three children (GLN245A, GLN244 and GLN258) is clustered in the west of the necropolis, and the parents share the same body position (on the left side), while the offspring all display the same body position (supine). In another example, a mother and her son (GLN225 and GLN226) display the same body position, and both had ochre in their burial. However, these observations were not systematic across the different nuclear families (Supplementary table 22, Extended Data Fig. 5a).

We also detected some similarities in the body position of fathers and sons being adults without offspring buried in the site: GLN237A and GLN221B are in the biggest graves lying down on their left side, with hyper-flexed arms and extended lower limbs. GLN275 and GLN216 are both on their left side, with hyper-flexed arms and legs.

Pedigree B

Some similar funerary practices are observed among the female individuals of Pedigree B (Supplementary table 22, Extended Data Fig. 3c). These individuals present similar decay space and grave good associations, even though this is not true for all individuals. Males do not share any common pattern across the four generations. Some degree of structure can be observed in the distribution of the burials. First generations appear on a north-east/south-west axis, while later generations are located on both sides of this axis, which seems to indicate some symmetry.

Isolated individuals

Since no clear pattern could be assigned to the different nuclear families, it is not possible to extrapolate or even suggest any link between the biologically unlinked and unrelated individuals with known families. For instance, it is expected that the seven unlinked adult females are partners of some of the adult males buried on the site, but in the absence of offspring, genomics can of course not detect these relationships. Unfortunately, the archaeological data (position, grave goods, spatial location) cannot help to propose more associations (Supplementary table 22, Extended Data Fig. 3c).

Supplementary Note 13. Bayesian modelling of radiocarbon dates
Stéphane Rottier

The radiocarbon dataset

Twenty-five radiocarbon dates have been previously published[37]. Eight new dates are available in this study, seven of which have been generated within the framework of the ANR/DFG-funded project INTERACT at the CEDAD - CEntro di DAtazione e Diagnostica, Salento University, Lecce, Italy (code LTL), and another, for GLN275, was generated at the CDRC - Centre de Datation par le RadioCarbone, Lyon 1 University, Lyon, France. The full range of all radiocarbon dates from Gurgy spans from 5,205 to 4,353 calibrated (cal.) BCE (according to IntCal20.14c[133]). All the details are available in Supplementary table 1.

We note some inconsistencies between the radiocarbon date estimates and the individuals within the order of generations on the reconstructed pedigrees, taking into account the estimated age at death and the biological relationships. For example, the radiocarbon date of GLN201 in generation 5 of pedigree A ranges from 5,205 to 4,839 cal. BCE while his father's (GLN202) radiocarbon date ranges from 4,534 to 4,353 cal. BCE (95%). Even if the son died before his father, it is impossible for such a chronological gap to occur between the two. This example as well as others reported recently[134] shows that biological relatedness can be taken into account to identify potential radiocarbon dating outliers.

Bayesian radiocarbon date modelling with ChronoModel 2.0.18

The genomic data obtained for Gurgy and the reconstruction of the pedigrees required a reassessment of the radiocarbon dataset available for the site. So far, in the absence of contextual observations that would help to constrain the calibrated radiocarbon dates, the full interval spanning from 5,204 to 4,356 cal. BCE has been used (Extended Data Fig. 10a). This is not entirely incorrect, but according to the new pedigrees, some dates must be considered outliers. Shifts of several hundred years in the chronological intervals for some individuals who are only separated by a few generations at most are simply impossible[134]. Moreover, spatial management, especially the rare overlaps, and the way they are almost always superimposed on the outline of the first dig, as well as the possible marking of grave sites[23], are all indications that already suggest a relatively short period of use for the site.

Chronological modelling based on date series, to which constraints are applied, have been extensively developed in the last few decades[134-136]. Using the Bayesian approach implemented in the ChronoModel software[137,138], it is possible to largely reduce the width of the chronological interval in which the Gurgy necropolis was in use.

ChronoModel 2.0.18 is a software package that aims to model archaeological data in order to estimate a date for a single event, or sequence of events, based on individual dates from archaeological artifacts assumed to be contemporaneous. The model is based on a hierarchical Bayesian statistical approach which includes the potential outliers that might be due to different errors (laboratory and calibration curve errors, contamination, taphonomy). Individual dates can be constrained by evidence from external, contextual observations such as stratigraphical or typochronological data. ChronoModel 2.0.18 is an open-source and freely-available software (https://chronomodel.com).

To better model the radiocarbon date estimates on the study interval (set between 5500 and 3500 BCE), we initialised a total of 33 events (radiocarbon dates) into 15 phases (pedigrees/groups of individuals), with 8 total phase constraints (see Supplementary table 25 for exact details). We then ran three independent Markov chain Monte Carlo chains, from

three different seed values. Using a mixing level of 0.99, for each chain we ran 1000 interations for burn-in with 20 batches, 500 iterations per batch, and a thinning parameter of 10, yielding a total of 111000 total iterations per chain. We used the default Metropolis-Hastings adaptive Gaussian Random Walk method for all events.

Relative chronology

First, it should be kept in mind that the chronological range resulting from radiocarbon dating is in a 95% confidence interval, and hence only includes 95% of the possible dates for the event being dated. The constraints that will be applied to the series of dates therefore theoretically allow us to find a new interval in which 95% of the possible dates for this event would be located. This event may be a one-off in time, or it may develop over a period of time, for which we will then speak of a phase.

The first step consists of a time constraint corresponding to the expected duration of use of the necropolis estimated by the number of generations of the bigger pedigree, which indicates a limit for the time of development of the phase in question.

Information from the pedigrees

The individual GLN270B is the common ancestor in Pedigree A. He was discovered in the only secondary burial, likely brought from another location where he was first buried, and where his body decomposed. He had at least three sons who had descendants themselves, some of whom were buried in Gurgy. Nine of the grandsons and one granddaughter reached adulthood and are also buried at the site. We can therefore hypothesize that it was this third generation which came to the site, leaving behind their siblings and ancestral relatives who had died before at a younger age.

The presence of subadults in the fourth generation corroborates this hypothesis, as they were the first subadults to die once the group had settled in Gurgy. However, the deficit of children in the fourth generation is notable (only 4 subadult individuals for 15 identified adults in addition to 14 adults who were inferred from the pedigrees but who are missing in our dataset). We suggest that the third generation came to the site with their children born elsewhere who then grew up around Gurgy. This would explain the low number of subadults buried in the site for this generation.

In the fifth generation, the ratio between the number of children buried (n=10) and the number of adults who would have been born *in situ* (n=9) is balanced. Nevertheless, of these adults born on site, only 5 are available on our dataset, which raises the question of whether the remaining individuals had left before their death. This echoes the exclusive presence of subadults in the sixth and seventh generations.

The second pedigree, Pedigree B, cannot help us to narrow the age estimates because it is impossible to link it to Pedigree A chronologically. However, the overall pattern is similar to Pedigree A. The last two generations are represented almost exclusively by subadults (with the exception of the adult female GLN277, descendant of the main lineage), whereas the first two are represented only by adults.

Therefore, if we use the Pedigree A, excluding the founding and migrating generations, and considering the possible gaps between generations, the duration of the site use was likely rather short, perhaps only 2-3 generations or ~56-84 years (we consider one generation to be 28 years[106]).

Following these observations, we considered the relationships established in Pedigree A and used these as a framework for constraints (Extended Data Fig. 10b).

Information from the archaeological data

In a second step, the constraints arising from stratigraphic relationships observed in the field were applied, as well as the coherent groupings between individuals who show without doubt anteriority over others, either individually or grouped together (Extended Data Fig. 10c).

For example, the four siblings of individual GLN317, specifically GLN212, GLN213, GLN224 and GLN255, were buried to the west of GLN317. However, the mother of their sons, GLN315 was buried to the east, and GLN223, his granddaughter, was buried on top of him. The other son of GLN317, GLN202, probably died later as he was buried in another part of the necropolis, together with other branches of the Pedigree A, and possibly the most recently deceased of this family line, including GLN201. The individuals GLN243A and GLN243B, buried in the same pit on the top of each other, clearly show the anteriority of GLN243B. Given their position in the pedigree, their age-at-death and their location in the necropolis, they can also be considered anterior to GLN201 (Extended Data Fig. 10c).

Results

Taking into account the constraints given by both the pedigrees and the archaeological context, we modelled five separate scenarios with ChronoModel 2.0.18. We modelled one scenario with a 15-year interval of use to test the hypothesis at a very low threshold, another two scenarios with a 30-year and a 60-year interval, and another scenario with an 80-year interval in order to test the sustainability of the model. We also investigated a scenario with a 120-year interval to explore the volatility of the model (Extended Data Fig. 10d).

For the 15-year interval scenario, the heterogeneity of the date set does not allow for the model to be applied, placing 95% of the possible dates in a range between 4,793 and 3,915 cal. BCE. The constraint applied here is thus too narrow to be feasible.

For a proposed duration of 30 years, the model calculates a possible effective duration of 27.5 to 30 years with a settlement starting between 4,731 and 4,637 cal. BCE and an abandonment between 4,704 and 4,610 cal. BCE (Extended Data Fig. 10d). For a duration of 60 years, the model estimates that the site was settled between 4,739 and 4,649 cal. BCE and abandoned between 4,681 and 4,591 cal. BCE (Extended Data Fig. 10d). For a duration of 80 years, the model calculates that the site was settled between 4,751 and 4655 cal. BCE and would be abandoned between 4,672 and 4,576 cal. BCE (Extended Data Fig. 10d). For a duration of 120 years, the start of use of the necropolis would be estimated to be between 4,761 and 4,667 cal. BCE and places its abandonment somewhere between 4,646 and 4,552 cal. BCE (Extended Data Fig. 10d).

Finally, irrespective of which interval we consider in the Bayesian modelling, from 30 to 120 years of duration of the necropolis, the pedigrees and archaeological context allow us to constrain the radiocarbon dates to an interval covering at most four centuries, and at the least two centuries, from the mid-48th century BCE to the mid-46th century BCE (Extended Data Fig. 10d).

Supplementary Note 14. Interpretation of the Gurgy necropolis and inferences on the settlement

Maïté Rivollat, Stéphane Rottier, Wolfgang Haak, Heidi Colleran, Marie-France Deguilloux, Léonie Rey, Gwenaëlle Goude, Vincent Balter

Supplementary Note 14.1. Social inferences

Patrilineality

Based on the reconstructed pedigrees A and B (as demonstrated in Supplementary Notes 2 and 3), we almost exclusively observe that each generation is linked to the previous generation through the biological father, which structures the whole group of Gurgy 'les Noisats' by the paternal lineage. This paternal lineage, characterized by the Y-chromosome haplogroup G2a2b2a1a2 (terminal SNP Z38302), is carried by 89% of the males of the group (Supplementary Note 7).

A patrilineal system, as shown by the genetic connections, was proposed before on the basis of archaeological features[40]. Here, the burials of fathers and their subadult male offspring are located significantly closer to each other than any other pairs of individuals (see p-values in Supplementary Note 12, Fig. 1e), even though we observe a general trend of spatial clusters following genetically closely related individuals (Fig. 1c).

The main paternal line of Pedigree A starts with individual GLN270B. This man has a particular archaeological status as his burial is the only secondary burial of the site, located within the grave of the female GLN270A, from whom we could not obtain DNA data (Fig. 1d). Only his long bones were deposited in the pit, likely in a bundle next to the articulated skeleton of GLN270A, while the rest of the skeleton of GLN270B was missing. This suggests that these remains have been transferred and buried during the early phase of the site, probably because he represented, together with his brother GLN231A, the main ancestors of the pedigree, and potentially the senior lineage male. This leads us to hypothesize that this grave was a founding event in the history of the necropolis, even though the relation to the female GLN270A remains unclear. GLN270A could be a close relative (mother, sister, daughter, or, possibly, a further connection), or reproductive partner. Alternatively, it could also be the burial of a random person, whose grave pit was prepared and to whom the remains of GLN270B were added opportunistically. Irrespective of the explanation, the will to translocate the remains of this main ancestor to the site, even if he had potentially died a long time before the secondary burial, marks the importance of this individual and his lineage in the creation of this new burial place for, and by, his descendants. If GLN270A was indeed the partner in life of GLN270B, then it is also interesting to note that the translocated remains were buried with her and not his brother (GLN231A) or his potential uncle/half-sibling GLN320, which would further underpin the significance of this lineage, now represented through her, and thus GLN237A potentially being the first-born son.

In fact, the importance of the main paternal lineage can also be traced in the subsequent generation directly following GLN270B. The two largest graves at the site were built for his son GLN237A, the only individual of his offspring who yielded DNA data, even though he must have had at least two more sons as inferred from the pedigrees who might have been buried at the site, but for whom no DNA data were obtained, as well as his grandson GLN221B, *i.e.,* the son of GLN237A (Fig. 1a, Extended Data Fig. 6d). We hypothesize that a form of social status was transmitted along the paternal line to his son and grandson, which is visible in some of the funerary practices.

Both genetic and archaeological data are consistent with a patrilineal structure of the group, potentially reflecting local understandings of genealogy and descent.

Patrilocality and female exogamic residential system

In parallel to the patrilineal signal, we also observe evidence for the practice of patrilocality within the group. Adult females buried at the site, whether they are mothers or not, are from a different lineage than those of the main pedigrees. Six out of twenty of these individuals represent an exception to this rule (GLN325, GLN212, GLN213, GLN277, GLN288 and GLN289B; Extended Data Fig. 2b), with only two out of these six having children buried on site. We note that almost all female descendants from the main lineage who reached an adult age are missing, if we account for a natural expected ratio of 1.05:1 males/females at birth[139], while the ratio among adult offspring at Gurgy is 4.5:1. This can in parts be explained by males staying in the group, *i.e.*, practices of patrilocality, akin to the patrilineal structure discussed above in conjunction with a female exogamy, in which females move from their birthplace to their reproductive partner's home. Seven adult females are not connected to the pedigrees, and also not distantly related as shown by the IBD sharing analysis (Supplementary Notes 3 and 5). We thus speculate that these could be the reproductive partners of males from the main pedigrees, with whom they either did not have offspring together, or whose offspring were not buried at the site, or for whom we did not recover DNA, that would allow us to link these females to the pedigrees. This pattern of female exogamy is clearly visible in the Extended Data Fig. 7a where a significant sex difference is observed with respect to the mean relatedness coefficient at the site, meaning that adult females had on average significantly fewer relatives at the site.

However, we also observe a strong imbalance between the number of adult males (n=38) and females (n=20) buried at the site. If we only consider the 42 reproductive unions inferred from the offspring buried at the site, the shortage of females (9 versus 20 males) is also striking, which means that a substantial proportion of adult females are missing in the necropolis. This suggests that it was twice as likely for an adult male to be buried at the site, than for a female at Gurgy, even though these females were an integral part of the group at a certain point in time, as they had offspring with local partners. This preferential funerary bias needs to be considered independently from the signal of female exogamy that we observe, but leaves open questions with respect to why these mothers were not buried at the site, where they went and where and if they were buried.

The data from Gurgy also allowed us to look more closely at the subadult individuals, as many of which are buried in the necropolis (n=37). The ratio of males to females of 1.06:1 matches the expected natural ratio of 1.05:1[139], meaning that the sex ratio of subadults had no preferential bias (Extended Data Fig. 7b). Looking at the age-at-death distribution, the vast majority of the subadults were younger than 15 years old (n=34), with most of the children younger than 8 years old (n=27) (Extended Data Fig. 2c). The difference in sex ratio between subadults and adults suggests that older daughters, from around at least the age of 15, and maybe slightly younger, had left to join a new group, in line with a female exogamic residential system.

This observation leads us to explore whether the age at which females left the group was related to a social threshold and tested this hypothesis by using different lines of evidence. On the basis of the archaeological context at Gurgy, we observe a shift in the funerary practices of children who died at around 7-8 years of age, when the usual grave goods accompanying the younger children were no longer used (Extended data Fig. 5b). An additional shift happened at around 15-16 years of age-at-death when they were associated with the same grave goods as adults, which could reflect the age of initiation or the rite of

passage, i.e. social threshold of accession to adulthood[108]. Sex-specific rites of passage at this age are well known, specifically related to the moment when specific social and gender roles are assigned and/or boys and girls are ritually separated[140]. This often involves differential access to sites and places and the introduction of tabooed or permitted foods, which could be detectable by stable isotopic analyses. However, carbon, nitrogen, sulphur isotope data highlight a significant dietary, sex-based difference in adults that could reflect a gender-biased access and/or differential treatment[36]. To explore this sex-based difference in subadults (Extended data Fig. 5c), we ran a cluster comparison using the Calinski-Harabasz Index and calculated the ratio of the sum of between-cluster dispersion and of inter-cluster dispersion for all clusters. The empirical p-value, calculated using a permutation test with all $\frac{17!}{9!8!} = 24{,}310$ permutations of the possible cluster labels, is significant (p=0.01069), and remains significant when including the "undetermined" individuals, meaning the same difference can be observed in the diets of males and females during childhood (Supplementary table 24, Extended data Fig. 5c).

Female genetic diversity and potential provenance

On the basis of the patterns of a female exogamic residential system employed by the Gurgy group, we explored the genetic diversity and the potential origin/provenance(s) of these females to gain an understanding of the social rules that governed this community. Heatmaps constructed from pairwise outgroup-f_3-statistics (n=16) and IBD sharing (n=12) (Supplementary Note 5, Extended Data Fig. 4) show very few connections between females. We detect three pairs of females who were related in the 3rd or 4th degree, while all other pairs are not genetically related, or too distantly to be detected, which corresponds to the expected background diversity of the population. The mitochondrial diversity in Gurgy reflects the mobility pattern (Supplementary Note 6), as the mothers of each generation contributed new mtDNA lineages and no mitochondrial haplogroup is transmitted further than one daughter/son generation, except for the female GLN325 who stayed in her lineage group and passed her mitochondrial haplogroup on to her offspring of the next generations. The analysis of runs of homozygosity (Supplementary Note 4, Extended Data Fig. 9c) also show an absence of inbreeding in the group. Only one individual, GLN282, has long ROH which correspond to him being the offspring of a 2nd or 3rd cousin relationship, but since his mother was not buried at Gurgy, or not successfully genotyped, we cannot position her precisely within Pedigree A. The overall level of background relatedness corresponds to a relatively medium-sized population, compared to the large size of LBK groups[64] and is consistent with the reconstructed pedigrees. Finally, the analysis of strontium (Sr) isotopes also shows that female partners, who are identified as exogenous in the pedigrees, had lower Sr values than their respective mates, a signal that is clearly visible and repeated in each subsequent generation (Supplementary Note 11), corroborating exogenous origin of the females in line with the genetic results. The independent lines of evidence suggest that the Gurgy community maintained a social system of female exogamy with a number of external groups, presumably bound to by reciprocal alliances, which may have been structured by a range of features, such as population size, access and exchanges of resources, network, or common linguistic and cultural affinities.

Patrilineal and patrilocal norms and exceptions

Assuming the existence of a female exogamic residential system with diverse origins of exogenous females as a baseline, we also observe a number of exceptions to this "norm" at different levels on the maternal line.

One exception to this main structure is the adult female GLN325, a descendant of the main lineage, whose partner, GLN275, is an individual who was genetically unrelated to the main lineage, and carried the only other Y-chromosome haplogroup of the site, H2m. GLN311, an adult male designated as "unlinked unrelated" in Figure 1a, also carried Y-chromosome haplogroup H2m, and is 2nd-degree related to GLN270B in the Pedigree A through the maternal side, but we are unable to characterize his exact relationship within the tree. The particular case of this second male lineage, with connections to different generations, but specifically via the adult female GLN325, implies a degree of flexibility to the norm as viewed from the two main pedigrees. The fact that the partner of female GLN325, a male from another lineage, was evidently integrated into the group and had offspring with a lineage female shows that he and/or his status was not seen to be in conflict with the dominant lineage for land access and/or other claims. This was possibly indirectly legitimized through the 2nd degree relationship of GLN311 with GLN270B, which links both lineages early in the tree. This could in turn indicate that the group was small in comparison, and therefore welcoming, in some situations, to incomers who were not direct descendants of the main founder lineage, or that some other social arrangements were involved, e.g., rights of *usufruct*[141] which gave access to individuals to use the land and/or resources, but not to own or inherit them.

Another exception to the observed predominant form of social organisation, inferred from the Gurgy funerary community, can be found in Pedigree B, and concerns female individual GLN288 and her daughter GLN289B. As the parents of GLN288 are missing, we cannot determine whether GLN288 is linked to the pedigree through her mother or her father. If she was linked through her mother to the main lineage of Pedigree B, we cannot exclude that the mother is missing because she had left the group to join another group as hypothesized for most of the other females from Gurgy, while her daughter GLN288 later returned to the group, then considered as an 'exogenous' partner. If she was linked through her father to Pedigree B, the main lineage is continued by one more generation. Irrespective of the two possibilities, GLN288 and GLN325 represent adult daughters, who had adult offspring buried at the site, which gives both of these individuals a special status. By contrast, the daughters GLN289B, GLN212 and GLN213 from Pedigree A represent the only three adult daughters without offspring. If we were to apply a strictly patrilocal and female exogamic system, these three should also have left to another group, therefore their presence at the site is also part of the exceptions observed in this cemetery.

Finally, the individual GLN282 with an inbreeding signal, resulting from his/her parents being 2nd or 3rd cousins also shows an exception to the rule by allowing rather closely related individuals to reproduce (Supplementary Note 4). The number of exceptions to the norm leads us to suggest that the patrilineal and patrilocal system was not absolutely strict or dogmatic social norm, but was rather permeable, allowing for exceptions or variations to the main inferences, for reasons we cannot assess with confidence.

Interaction with the regional network and size of the population

Results from the IBD sharing analysis also revealed additional connections beyond the reconstructed pedigrees, indicating links through female lines. Here, we observed several pairs of the pedigrees that appear to share more IBD than expected according to the reconstructed pedigrees, but without questioning its robustness (Supplementary Note 3, Fig. 2). This includes individuals within Pedigree A, but also between Pedigrees A and B. All detected links, although relatively distant, can only be explained through the female lines. A likely explanation is a scenario in which female descendants of the woman who had left the Gurgy community returned after a few generations to find a partner in Gurgy in return.

Alternatively, incoming female partners came from the same community and were maternally related. Both cases would imply that the network of reciprocal mobility within which these movements happened, was small to retain continuous connections (intentional or unintentional), but at the same time also sufficiently large to integrate entirely unrelated females. This observation is consistent with the results from ROH for the entire group, which show intermediate levels of background relatedness that correspond to a medium-sized meta-population (Supplementary Note 4). Together, these findings lend support to the existence of a wider and more fluid exchange network of many, potentially smaller groups, a phenomenon which is called 'generalized exchange' in ethnographic studies[142].

Monogamous pairing

The absence of half-sibling relationships in the group of Gurgy (Supplementary Note 2) is another aspect that is critical for the inference of social structure of prehistoric communities. This observed absence is striking as this would imply that polygamy was not a common practice in this community. It also rules out serial monogamy, *i.e.,* a second or third partnership after the death of the first partner, at least based on the individuals buried at the site, which as a result lends support to exclusively monogamous pairings.

Inferences beyond the site

From an archaeological perspective, the site Gurgy stands in contrast to the regional context of contemporaneous monumental sites that are associated with the Cerny culture, and which were built for selected individuals[12]. The only monumental site from the Cerny area which has been genetically investigated to date, Fleury-sur-Orne in Normandy, shows a strong social selection of individuals buried in different monuments according to different patrilineal lineages[22] (Supplementary Note 1). Given the structural differences when compared to Gurgy, but also to STP sites from the Paris Basin, direct inferences cannot made.

One of the possible interpretations for the situation at Gurgy is that two different communities, with different funerary practices and attitudes towards social ranking, were co-existing within the same territory at the same time. This hypothesis would find support from a cultural point of view, as Gurgy does not show a clear attribution to the Cerny culture based on archaeological data, although the site is contemporaneous, and Cerny sites are located nearby. Gurgy burials indeed show multiple influences from different contemporaneous cultural groups, some local, such as alcove burials from LBK-derived groups in the West, with others originating further away, such as the Chamblandes cists from the Alps, or the presence of specific shell types from the Mediterranean area (Supplementary Note 1). These diverse cultural influences question the representativeness of Gurgy in the local Cerny context, but also of its social practices. However, the assumption that Gurgy was not fully culturally embedded in the surrounding context does not align with findings from the genetic data, which show strong links within a wider, but not completely random, biological network over several generations.

Given the absence of evidence that would point to a selection of individuals, *i.e.* an elite, on the basis of sex, age, economic or social hierarchies at the regional level, an alternative interpretation would be that the site represents the burial practices of the non-elite, that is of a broader stratum of a society in which the STP-buried individuals represent the elite at the regional level. Considering this hypothesis, and the fact that Gurgy was used by a single genetically related group, we would expect contemporaneous graveyards of similar sizes to meet the expectations from the observed genetic diversity estimates, such as

mitochondrial haplogroup diversity and runs of homozygosity. However, only three other contemporaneous graveyards without monuments are known from the area, all of which are also much smaller: Monéteau 'Macherin' located only three kilometres away from Gurgy, Vignely 'La Porte aux Bergers', and Chichery 'sur les Pâtureaux' with 15, 17, and 27 buried individuals, respectively[16-18,20] (Supplementary Note 1). If we consider all of the individuals buried at these sites as potential non-elite people, the total number (n=187) is too low compared to the known 120 individuals buried in the monumental sites[12,13,20], and would either suggest a substantial funerary bias (a higher chance of not being granted a burial) or an excavation bias (a higher chance of not observing regular inhumations, or finding them).

Looking at the site level, we can observe a slight emphasis on, if not at least a hierarchy of, some individuals. For example, the larger sizes of the graves of GLN270B's son (GLN237A) and grandson (GLN221B). However, these hints are far from the ostentatious demonstration visible in the STPs, and none of the elements that emphasize the hierarchical structure at STP sites, such as monuments, gender-related scenography, grave goods, are visible at Gurgy.

The translocation and secondary inhumation of individual GLN270B, the main ancestor of the large Pedigree A, points strongly to a specific funerary treatment compared to all other individuals buried at Gurgy, even though no other sign of power/wealth in the material culture was found. We speculate that it must have been important for his community/family to establish a connection to the ancestors and to (re)bury him as a founder in the very place where also his descendant will be buried for a few generations. However, this did not reach a level of supra-regional significance outside the Gurgy community, as no externally visible signs of representation were found.

At present, we lean towards the last hypothesis, which suggests that the different necropolises without monuments in the Paris Basin do represent the 'commoners', while many others, which we assume would have had a similar structure and/or organisation, have not been found yet. We argue that this scenario is more in line with the genetic data that shows evidence for wider and well-connected network over several generations. However, we acknowledge that the lack of comparative data from STP sites and non-monumental sites from the same region presents a current limitation to go beyond this tentative interpretation.

Supplementary Note 14.2. Demographic inferences

A group structure as described above is a perfect dataset to test paleodemographic hypotheses. Following the demographic approach proposed by P. Sellier (2011)[143], we first tried to characterize the population sample under study. When we construct the mortality table from the ages at death of all the individuals in the necropolis (Supplementary table 19), we notice a deficit in infants/newborns ([0] year class) compared to an expected pattern of mortality in (pre-)history[144], which is common for this period (Extended Data Fig. 6f). On one hand, when we look at the Pedigree A, the near absence of any young children is obvious in the first generations, as is the absence of adults in the last generations. It may seem reasonable to consider that some of the individuals definitely missing in generations 2 and 3 could be part of the individuals buried here, but for whom we have no genetic data. On the other hand, it is possible that for generation 2 and from generation 4 onwards the missing adults did not die on site.

Therefore, the structure of this population does not follow a natural mortality curve, or rather the composition of the buried group indicates a selection against natural mortality (Extended data Fig. 6f). The selection concerns the absence of children in the early stages, and then the absence of adults towards the end of the occupation phase. For the generations in between, an absence of adult women from the patrilocal lineage is noticeable, while the buried women come from other lineages, that are themselves rarely related to each other's.

In generations 2 and 3 of Pedigree A, we observe that four couples had four or five sons and two of these couples one or two more daughters, all of whom reached adulthood. As the majority of these children in generations 3 and 4 are men who died in adulthood, buried or not at the site, we would expect an equivalent number of women, as the ratio at birth between males and females is 1.05:1[139]. This suggests that each of these four couples must have had up to 10 or 12 children, and possibly more. Indeed, it can be considered that some of the individuals died at an early age, as expected from the mortality pattern commonly observed in prehistoric societies, but this cannot explain the absence of female individuals. Moreover, there are individuals in the necropolis who did not yield sufficient DNA data, and who are potentially these missing siblings.

There are therefore too many missing individuals to be able to construct new mortality tables by generation or according to other breakdowns, but, in spite of this, it seems interesting to suggest a possible range of numbers constituting the group using the Gurgy necropolis and the assumed living people simultaneously. To do this, we have to assume that all of the deceased individuals are present in this necropolis, which is false, as just stated. However, this *a priori* will allow us to make an *a minima* proposal. The equation proposed by Acsádi and Nemeskéri in 1970[145] could be used here:

$$P = k + \frac{De_0^0}{t}$$

where *P* represents the population size, here the group who lived in a same place at the same time, *D* the total number of deaths, e_0^0 the life expectancy at birth, *t* the time of use of the final site, and *k*, which is a correction coefficient set at 10% of *t*.

An attempt to calculate life expectancy at birth is provided in the mortality table (Supplementary table 19). These values must be taken with caution since at least the deceased subadults for generations 2 to 4 and most of the adults in generations 5 and 6 are missing. According to the estimates provided by the radiocarbon modelling, we can apply alternative estimates of the duration of site use.

Therefore, the calculation would be as follows:

- For a 15-year duration of use: *P* = 1.5+(128x33.1)/15 = 284 individuals.
- For a 30-year duration of use: *P* = 3+(128x33.1)/30 = 144 individuals.
- For a 60-year duration of use: *P* = 6+(128x33.1)/60 = 77 individuals.
- For a 120-year duration of use: *P* = 12+(128x33.1)/120 = 47 individuals.

If we set a lower life expectancy at birth, which would seem to be expected given the deficit of infants, then the number of individuals would also be lower, around 110 for a 30-year period of use and 59 for a 60-year period of use (214 for 15 years), and a life expectancy at birth of 25 years.

The estimate of a community of a few dozen people, ranging from 50 to nearly 200 is a credible range. The main drawback of this kind of approach is that any attempt to assess demographics on such data assumes a constant population[143], which is far from being the case with female arrivals and departures in particular, even if one could propose that these exchanges might compensate for each other. As we have demonstrated, the Gurgy community started with a small group, which founded the site, and expanded after one or two generations. This chronological dynamic, possibly fluctuating over time, also needs to be taken into consideration when we estimate the size of this group.

Supplementary Note 14.3. Local mobility inferences from multiple lines of evidence

As already described (Supplementary Note 13), the site of Gurgy 'les Noisats', especially when considering the findings from Pedigree A, indicates that the individuals that were buried at the site came from another geographic location, settled in the area, and used the necropolis over several (2-3) generations (Supplementary Note 13), before leaving for another settlement. The Sr isotope data support this scenario with lower $^{87}Sr/^{86}Sr$ values for the first generations, as a signal of their exogenous origin (Supplementary Note 11), similar to that of exogenous females.

Strontium isotope data revealed regular variations between each generation (Fig. 3). Keeping in mind the potential gaps between the generations, and the fact that individuals of each generation are not necessarily synchronous, it nonetheless seems that each generation settled in an area different from the previous one. The range of these territories is unknown, as Sr values are sensitive to subtle differences in the substratum, and the size of these territories could range from a few hundred meters to several (tens of) kilometers. This 'intergenerational territorial mobility' (Supplementary Note 11) is highlighted for a Neolithic group for the first time and attests to a "short-term" exploitation of the environment.

Carbon, nitrogen, and sulphur data

This "intergenerational territorial mobility", where each generation of individuals resides in a new location compared to the last, as depicted by Sr isotopes, is related to another important assessment: the variability of carbon, nitrogen, and sulphur (CNS) stable isotope ratios recorded in bones and teeth. Previous analyses of CNS stable isotope ratios in bone and dentine collagen have been performed on most of the individuals from Gurgy, highlighting age-, and sex-related variability[32,36]. Isotopic similarities were found to be linked to spatial proximity (groups of individuals buried in nearby graves shared more similar isotopic values, especially for N isotopes). The CNS isotopic variability recorded at the intra- and inter-individual level can be due to the territorial variability between the generations or family groups. The importance of environmental variability in stable isotope data for Neolithic humans and animals has already been demonstrated by Goude and Fontugne[146], but if an "intergenerational territorial mobility" is a common practice in agropastoral groups, the CNS isotopic ratios should be considered according to these new cultural and economic factors, rather than with a basic interpretative approach (involving age, sex, general biological/archaeological factors). The association of genetic information with CNS isotopic data reveals similarities between close family members. It is possible that individuals from a given family branch and close generations share similar diets, resources, and environments, demonstrating resource management strategies at the sub-lineage level. Therefore, the interpretation of isotopic data ($^{87}Sr/^{86}Sr$ and CNS) and, in turn, of human behaviors, changes with the additional evidence of biological relatedness, generations and status (such as genetically exogenous or unrelated people).

Consequently, the isotopic data of the Gurgy funerary assemblage provide new information on the behavior of early European farmers. Isotopic data from other European Neolithic groups clearly show a high (seasonal, multi-annual, or at least regular during infancy and childhood) mobility of Neolithic and prehistoric farmers in general[36,147]. This mobility is likely essential in adapting to the environment and to surviving, even within a context of plant/animal domestication.

An alternative model of mobility during the Neolithic

Evidence of mobility is visible in the Gurgy dataset, at least from a genomic point of view. Specifically, we observed evidence of mobility among women who relocated between groups, whereas men remained in their group (Supplementary Note 14.1). This pattern is clear in Gurgy, and we suggest that such a model must be extended to a regional network level to make this pattern consistent and sustainable between groups. The group at Gurgy came from some other location to use this necropolis, stayed for a few decades, and left for somewhere else (Supplementary Note 12).

The additional information provided by the isotopic analyses ($^{87}Sr/^{86}Sr$ and CNS) indicates another, more fine-grained level of mobility, on the basis of subtle evidence for a different environmental background for each generation. The alternative model suggests a move of each generation to another geographical location, potentially very close to the previous one, yet resulting in a slightly different isotope signature, while maintaining a common burial ground. The absence of settlements in the Gurgy area means that we cannot corroborate this hypothesis with archaeological data. The Gurgy necropolis is certainly central to the Gurgy community and represents the point of overlap for the two large pedigrees and associated kin, even though, as the isotopic data suggest, the people from Gurgy might not have lived consistently at the same place for each generation.

Seasonally changing residences, as reflected by the availability of natural resources, is a pattern that we are more used to observing in mobility studies of hunter-gatherers[148,149]. This study proposes a new outlook on the structure and organization of Neolithic subsistence, advocating a higher level of generational mobility (and/or fraction of the communities) than previously anticipated, but in a different mode than is known for hunter-gatherers.

References

1 Allard, P. The Mesolithic - Neolithic transition in the Paris Basin: a review. *Proceedings of the British Academy* **144**, 211-223 (2007).

2 Gallay, A. *Cistes de type Chamblandes: 15 ans de recherches, quels progrès?* , (Cahiers d'archéologie romande et Société préhistorique française, 2007).

3 Rottier, S., Mordant, C., Chambon, P. & Thevenet, C. Découverte de plus d'une centaine de sépultures du Néolithique moyen à Gurgy, les Noisats (Yonne). *Bulletin de la Société préhistorique française* **102**, 641-645 (2005).

4 Thevenet, C. *Des faits aux gestes... des gestes aux sens?: pratiques funéraires et société durant le Néolithique ancien en Bassin parisien.* (thèse de doctorat, Paris 1, 2010).

5 Constantin, C., Mordant, D. & Simonin, D. *La culture de Cerny. Nouvelle économie, nouvelle société au Néolithique. Actes du Colloque International de Nemours, 9-10-11 mai 1994*. Vol. 6 (Mémoires du Musée de Préhistoire d'Île-de-France, 1997).

6 Richard, G., Jagu, D., Guillon, F. & Girard, C. La sépulture des "Marsaules", Malesherbes (Loiret), et les sépultures sous dalle du groupe Essonne-Juine. *Revue archéologique du Loiret* **12**, 15-34 (1986).

7 Simonin, D. Premières données sur la nécropole des Fiefs à Orville (Loiret) et remarques à propos de la culture de Cerny. *Bulletin de la Société Archéologique, Scientifique et Littéraire du Vendômois*, 53-68 (1991).

8 Simonin, D., Bach, S., Richard, G. & Vintrou, J. Les sépultures sous dalle de type Malesherbes et la nécropole d'Orville. *Mémoires du Musée de préhistoire d'Ile-de-France*, 341-379 (1997).

9 Lichardus-Itten, M. Premières influences méditerranéennes dans le Néolithique du Bassin parisien, contribution au débat. in *Le Néolithique de la France. Hommage à Gérard Bailloud* (eds Jean-Paul Demoule & Jean Guilaine) 147-160 (Picard, 1986).

10 Sidéra, I. De mains méridionales en mains septentrionales. Le long transit des objets et des savoir-faire en Europe occidentale, vers 5100 av. J.-C. *Mélanges de la Casa de Velázquez. Nouvelle série* **40**, 17-32 (2010).

11 Moinat, P. & Chambon, P. *Les cistes de Chamblandes et la place des coffres dans les pratiques funéraires du Néolithique moyen occidental. Actes du colloques de Lausanne, 12 et 13 mai 2006.* (Cahier d'archéologie romande 110 et Société préhistorique XLIII, 2007).

12 Chambon, P. & Thomas, A. The first monumental cemeteries of western Europe : the „Passy type" necropolis in the Paris basin around 4500 BC. *Journal of Neolithic Archaeology* **12** (2010). https://doi.org:10.12766/jna.2010.37

13 Chambon, P. Revoir Passy à la lumière de Balloy: les nécropoles monumentales Cerny du bassin Seine-Yonne. *Bulletin de la Société préhistorique française* **100**, 505-515 (2003).

14 Sidéra, I. Le mobilier en matières dures animales en milieu funéraire Cerny: symbolisme et socio-économie. in *La culture de Cerny, nouvelle économie, nouvelle société au Néolithique. Actes du Colloque International de Nemours 9-10-11 mai 1994* (eds Claude Constantin, Daniel Mordant, & Daniel Simonin) 499-513. (1997).

15 Chambon, P. & Pétillon, J.-M. Des chasseurs Cerny ? *Bulletin de la Société préhistorique française* **106**, 761-783 (2009).

16 Augereau, A. & Chambon, P. *Les occupations néolithiques de Macherin à Monéteau (Yonne)*. Vol. Collection Mémoires, 53 (Société préhistorique française, 2011).

17 Chambon, P. *et al.* La nécropole du Néolithique moyen de Sur les Pâtureaux à Chichery (Yonne). *Gallia Préhistoire–Préhistoire de la France dans son contexte européen* **52**, 117-192 (2010).
18 Chambon, P. & Lanchon, Y. Les structures sépulcrales de la nécropole de Vignely (Seine-et-Marne). *Mémoires de la Société préhistorique française* **33**, 159-173 (2003).
19 Ghesquière, E. *et al.* Monumental cemeteries of the 5th millenium BC: The Fleury-sur-Orne contribution. *Megaliths – Societies – Landscapes, Early Monumentality and Social Differentiation in Neolithic Europe* **1**, 177-191 (2019).
20 Thomas, A. *Identités funéraires, variants biologiques et facteurs chronologiques : une nouvelle perception du contexte culturel et social du Cerny (Bassin parisien, 4700-4300 avant J.-C.)*. (thèse de doctorat, Bordeaux 1, 2011).
21 Desloges, J. Les premières architectures funéraires de Basse-Normandie. in *La culture de Cerny, nouvelle économie, nouvelle société au Néolithique. Actes du Colloque International de Nemours, 9-10-11 mai 1994* Vol. 6 (eds Claude Constantin, Daniel Mordant, & Daniel Simonin) 515-539 (Mémoires du Musée de Préhistoire d'Île-de-France, 1997).
22 Rivollat, M. *et al.* Ancient DNA gives new insights into a Norman Neolithic monumental cemetery dedicated to male elites. *PNAS* **119**, e2120786119 (2022).
23 Rottier, S. L'architecture funéraire des sépultures du Néolithique moyen des Noisats à Gurgy (Yonne, France). in *Les cistes de Chamblandes et la place des coffres dans les pratiques funéraires du Néolithique moyen occidental. Actes du colloque de Lausanne, 12 et 13 mai 2006* (eds Patrick Moinat & Philippe Chambon) 99-107 (Cahiers d'archéologie romande et Société préhistorique française, 2007).
24 Jeunesse, C. *Pratiques funéraires au néolithique ancien: sépultures et nécropoles des sociétés danubiennes (5500-4900 av. J.-C.)*. (Editions Errance, 1997).
25 Rottier, S. Observations préliminaires à l'étude des remplissages des tombes du Néolithique moyen I de Gurgy «Les Noisats»(Yonne). *Revue archéologique de l'Est* **55**, 279-285 (2006).
26 Schmitt, A. Une nouvelle méthode pour estimer l'âge au décès des adultes à partir de la surface sacro-pelvienne iliaque. *Bulletins et Mémoires de la Société d'Anthropologie de Paris* **17**, 89-101 (2005).
27 Moorrees, C. F. A., Fanning, E. A. & Hunt, E. E. Age Variation of Formation Stages for Ten Permanent Teeth. *Journal of Dental Research* **42**, 1490-1502 (1963). https://doi.org:10.1177/00220345630420062701
28 Scheuer, L. & Black, S. *Developmental Juvenile Osteology*. (Elsevier, 2000).
29 Brůžek, J. A method for visual determination of sex, using the human hip bone. *American Journal of Physical Anthropology* **117**, 157-168 (2002). https://doi.org:10.1002/ajpa.10012
30 Brůžek, J., Santos, F., Dutailly, B., Murail, P. & Cunha, E. Validation and reliability of the sex estimation of the human os coxae using freely available DSP2 software for bioarchaeology and forensic anthropology. *American Journal of Physical Anthropology* **164**, 440-449 (2017). https://doi.org:https://doi.org/10.1002/ajpa.23282
31 Murail, P., Bruzek, J. & Braga, J. A new approach to sexual diagnosis in past populations. Practical adjustments from Van Vark's procedure. *International Journal of Osteoarchaeology* **9**, 39-53 (1999). https://doi.org:10.1002/(SICI)1099-1212(199901/02)9:1<39::AID-OA458>3.0.CO;2-V
32 Rey, L., Salazar-García, D. C., Santos, F., Rottier, S. & Goude, G. A multi-isotope analysis of Neolithic human groups in the Yonne valley, Northern France: insights

into dietary patterns and social structure. *Archaeological and Anthropological Sciences* **11**, 5591-5616 (2019).
33 Duday, H. *The archaeology of the dead: lectures in archaeothanatology*. (Oxbow, 2009).
34 Le Luyer, M., Coquerelle, M., Rottier, S. & Bayle, P. Internal Tooth Structure and Burial Practices: Insights into the Neolithic Necropolis of Gurgy (France, 5100-4000 cal. BC). *PLOS ONE* **11**, e0159688 (2016). https://doi.org:10.1371/journal.pone.0159688
35 Rey, L., Goude, G. & Rottier, S. Comportements alimentaires au Néolithique: nouveaux résultats dans le Bassin parisien à partir de l'étude isotopique (δ13C, δ15N) de la nécropole de Gurgy «Les Noisats»(Yonne, Ve millénaire av. J.-C.). *Bulletins et Mémoires de la Société d'Anthropologie de Paris* **29**, 54-69 (2017).
36 Rey, L., Rottier, S., Santos, F. & Goude, G. Sex and age-related social organization in the Neolithic: A promising survey from the Paris Basin. *Journal of Archaeological Science: Reports* **38**, 103092 (2021).
37 Rivollat, M. *et al.* When the Waves of European Neolithization Met: First Paleogenetic Evidence from Early Farmers in the Southern Paris Basin. *PLoS One* **10**, e0125521 (2015). https://doi.org:10.1371/journal.pone.0125521
38 Rivollat, M. *et al.* Investigating mitochondrial DNA relationships in Neolithic Western Europe through serial coalescent simulations. *European journal of human genetics* (2016).
39 Rivollat, M. *et al.* Ancient genome-wide DNA from France highlights the complexity of interactions between Mesolithic hunter-gatherers and Neolithic farmers. *Science Advances* **6**, eaaz5344 (2020).
40 Le Roy, M. *et al.* Distinct ancestries for similar funerary practices? A GIS analysis comparing funerary, osteological and aDNA data from the Middle Neolithic necropolis Gurgy "Les Noisats"(Yonne, France). *Journal of Archaeological Science* **73**, 45-54 (2016).
41 Kocher, A. *et al.* Ten millennia of hepatitis B virus evolution. *Science* **374**, 182-188 (2021). https://doi.org:10.1126/science.abi5658
42 Monroy Kuhn, J. M., Jakobsson, M. & Günther, T. Estimating genetic kin relationships in prehistoric populations. *PLOS ONE* **13**, e0195491 (2018). https://doi.org:10.1371/journal.pone.0195491
43 Lipatov, M., Sanjeev, K., Patro, R. & Veeramah, K. R. Maximum Likelihood Estimation of Biological Relatedness from Low Coverage Sequencing Data. *bioRxiv*, 023374 (2015). https://doi.org:10.1101/023374
44 Weir, B. S., Anderson, A. D. & Hepler, A. B. Genetic relatedness analysis: modern data and new challenges. *Nature Reviews Genetics* **7**, 771-780 (2006). https://doi.org:10.1038/nrg1960
45 Rohrlach, A. B., Tuke, J., Popli, D. & Haak, W. BREADR: An R Package for the Bayesian Estimation of Genetic Relatedness from Low-coverage Genotype Data. *bioRxiv*, 2023.2004.2017.537144 (2023). https://doi.org:10.1101/2023.04.17.537144
46 Villalba-Mouco, V. *et al.* Genomic transformation and social organization during the Copper Age–Bronze Age transition in southern Iberia. *Science Advances* **eabi7038** (2021).
47 Villanueva, R. A. M. & Chen, Z. J. ggplot2: elegant graphics for data analysis. (2019).
48 Wickham, H. *et al.* Welcome to the Tidyverse. *Journal of open source software* **4**, 1686 (2019).
49 Bache, S. M. & Wickham, H. A Forward-Pipe Operator for R. *Package R* (2016).

50 Dowle, M. *et al.* "Package 'data. table'." Extension of 'data. frame 596. (2019).
51 Firke, S. Janitor: Simple tools for examining and cleaning dirty data. R package version 2.0. 1. *Computer software]. https://CRAN. R-project. org/package= janitor* (2020).
52 Rubinacci, S., Ribeiro, D. M., Hofmeister, R. J. & Delaneau, O. Efficient phasing and imputation of low-coverage sequencing data using large reference panels. *Nature Genetics* **53**, 120-126 (2021). https://doi.org:10.1038/s41588-020-00756-0
53 Mathieson, I. *et al.* Genome-wide patterns of selection in 230 ancient Eurasians. *Nature* **528** (2015).
54 1000 Genomes Project Consortium *et al.* A global reference for human genetic variation. *Nature* **526**, 68-74 (2015). https://doi.org:10.1038/nature15393
55 Ringbauer, H. *et al.* ancIBD - Screening for identity by descent segments in human ancient DNA. *BioRxiv* (2023).
56 Ringbauer, H., Novembre, J. & Steinruecken, M. Parental relatedness through time revealed by runs of homozygosity in ancient DNA. *Nature Communications* **12** (2021). https://doi.org:doi.org/10.1038/s41467-021-25289-w
57 Fernandes, D. M. *et al.* A genetic history of the pre-contact Caribbean. *Nature* **590**, 103-110 (2021). https://doi.org:10.1038/s41586-020-03053-2
58 Childebayeva, A. *et al.* Population Genetics and Signatures of Selection in Early Neolithic European Farmers. *Molecular Biology and Evolution* **39**, msac108 (2022). https://doi.org:10.1093/molbev/msac108
59 Arzelier, A. *et al.* Neolithic genomic data from Southern France showcase intensified interactions with hunter-gatherer communities. *IScience* **25**, 195387 (2022). https://doi.org:https://doi.org/10.1016/j.isci.2022.105387
60 Patterson, N. *et al.* Ancient admixture in human history. *Genetics* **192**, 1065-1093 (2012).
61 Warnes, G. R. *et al.* gplots: Various R programming tools for plotting data, R package version 2.4.1. (2019).
62 Maricic, T., Whitten, M. & Pääbo, S. Multiplexed DNA Sequence Capture of Mitochondrial Genomes Using PCR Products. *PLOS ONE* **5**, e14004 (2010). https://doi.org:10.1371/journal.pone.0014004
63 Haak, W. *et al.* Massive migration from the steppe was a source for Indo-European languages in Europe. *Nature* **552**, 207-211 (2015).
64 Li, H. *et al.* The Sequence Alignment/Map format and SAMtools. *Bioinformatics* **25**, 2078-2079 (2009). https://doi.org:10.1093/bioinformatics/btp352
65 Andrews, R. M. *et al.* Reanalysis and revision of the Cambridge reference sequence for human mitochondrial DNA. *Nature genetics* **23**, 147-147 (1999).
66 Peltzer, A. *et al.* EAGER: efficient ancient genome reconstruction. *Genome biology* **17**, 60 (2016).
67 Kearse, M. *et al.* Geneious Basic: an integrated and extendable desktop software platform for the organization and analysis of sequence data. *Bioinformatics* **28**, 1647-1649 (2012).
68 Weissensteiner, H. *et al.* HaploGrep 2: mitochondrial haplogroup classification in the era of high-throughput sequencing. *Nucleic Acids Research* **44**, W58-W63 (2016). https://doi.org:10.1093/nar/gkw233
69 Oota, H., Settheetham-Ishida, W., Tiwawech, D., Ishida, T. & Stoneking, M. Human mtDNA and Y-chromosome variation is correlated with matrilocal versus patrilocal residence. *Nature genetics* **29**, 20-21 (2001).
70 van Oven, M. & Kayser, M. Updated comprehensive phylogenetic tree of global human mitochondrial DNA variation. *Human Mutation* **30**, E386-E394 (2009).

71 Fowler, C. *et al.* A high-resolution picture of kinship practices in an Early Neolithic tomb. *Nature* (2021). https://doi.org:10.1038/s41586-021-04241-4
72 Rohrlach, A. B. *et al.* Using Y-chromosome capture enrichment to resolve haplogroup H2 shows new evidence for a two-Path Neolithic expansion to Western Europe. *Scientific Reports*, 11:15005 (2021). https://doi.org:doi.org/10.1038/s41598-021-94491-z
73 Feldman, M. *et al.* Late Pleistocene human genome suggests a local origin for the first farmers of central Anatolia. *Nature communications* **10**, 1218 (2019).
74 Lipson, M. *et al.* Parallel palaeogenomic transects reveal complex genetic history of early European farmers. *Nature* **551**, 368 (2017). https://doi.org:10.1038/nature24476
75 Marchi, N. *et al.* The genomic origins of the world's first farmers. *Cell* **185**, 1842-1859.e1818 (2022). https://doi.org:https://doi.org/10.1016/j.cell.2022.04.008
76 Cassidy, L. M. *et al.* A dynastic elite in monumental Neolithic society. *Nature* **582**, 384-388 (2020). https://doi.org:10.1038/s41586-020-2378-6
77 Brace, S. *et al.* Ancient genomes indicate population replacement in Early Neolithic Britain. *Nature Ecology & Evolution* **3**, 765-771 (2019). https://doi.org:10.1038/s41559-019-0871-9
78 Olalde, I. *et al.* The Beaker phenomenon and the genomic transformation of northwest Europe. *Nature* **555**, 190-196 (2018). https://doi.org:10.1038/nature25738
79 Gnirke, A. *et al.* Solution hybrid selection with ultra-long oligonucleotides for massively parallel targeted sequencing. *Nature biotechnology* **27**, 182-189 (2009).
80 Barquera, R. *et al.* Origin and health status of first-generation Africans from early colonial Mexico. *Current Biology* **30**, 2078-2091 (2020).
81 Immel, A. *et al.* Analysis of genomic DNA from medieval plague victims suggests long-term effect of Yersinia pestis on human immunity genes. *Molecular biology and evolution* **38**, 4059-4076 (2021).
82 Szolek, A. *et al.* OptiType: precision HLA typing from next-generation sequencing data. *Bioinformatics* **30**, 3310-3316 (2014).
83 Cao, K. *et al.* Analysis of the frequencies of HLA-A, B, and C alleles and haplotypes in the five major ethnic groups of the United States reveals high levels of diversity in these loci and contrasting distribution patterns in these populations. *Human immunology* **62**, 1009-1030 (2001).
84 Gonzalez-Galarza, F. F. *et al.* Allele frequency net database (AFND) 2020 update: gold-standard data classification, open access genotype data and new query tools. *Nucleic acids research* **48**, D783-D788 (2020).
85 Yunis, E. J. *et al.* Inheritable variable sizes of DNA stretches in the human MHC: conserved extended haplotypes and their fragments or blocks. *Tissue antigens* **62**, 1-20 (2003).
86 Immel, A. *et al.* Genome-wide study of a Neolithic Wartberg grave community reveals distinct HLA variation and hunter-gatherer ancestry. *Communications biology* **4**, 1-10 (2021).
87 Cassidy, L. M. *et al.* Neolithic and Bronze Age migration to Ireland and establishment of the insular Atlantic genome. *Proceedings of the National Academy of Sciences* **113**, 368 (2016). https://doi.org:10.1073/pnas.1518445113
88 Singh, R., Kaul, R., Kaul, A. & Khan, K. A comparative review of HLA associations with hepatitis B and C viral infections across global populations. *World journal of gastroenterology: WJG* **13**, 1770 (2007).
89 Thio, C. L. *et al.* Class II HLA alleles and hepatitis B virus persistence in African Americans. *The Journal of infectious diseases* **179**, 1004-1006 (1999).

90 Zhu, M. *et al.* Fine mapping the MHC region identified four independent variants modifying susceptibility to chronic hepatitis B in Han Chinese. *Human Molecular Genetics* **25**, 1225-1232 (2016).
91 Zhang, Y., Zhao, F., Lan, L., Qin, Z. & Jun, L. Correlation of HLA-DQB1 gene polymorphism of Xinjiang Uygur with outcome of HBV infection. *International Journal of Clinical and Experimental Medicine* **8**, 6067 (2015).
92 Li, X. *et al.* The influence of HLA alleles and HBV subgenotyes on the outcomes of HBV infections in Northeast China. *Virus research* **163**, 328-333 (2012).
93 van de Loosdrecht, M. *et al.* Pleistocene North African genomes link Near Eastern and sub-Saharan African human populations. *Science* **360**, 548-552 (2018). https://doi.org:10.1126/science.aar8380
94 Chaitanya, L. *et al.* The HIrisPlex-S system for eye, hair and skin colour prediction from DNA: Introduction and forensic developmental validation. *Forensic Science International: Genetics* **35**, 123-135 (2018). https://doi.org:https://doi.org/10.1016/j.fsigen.2018.04.004
95 Walsh, S. *et al.* Global skin colour prediction from DNA. *Human Genetics* **136**, 847-863 (2017). https://doi.org:10.1007/s00439-017-1808-5
96 Walsh, S. *et al.* Developmental validation of the HIrisPlex system: DNA-based eye and hair colour prediction for forensic and anthropological usage. *Forensic Science International: Genetics* **9**, 150-161 (2014). https://doi.org:https://doi.org/10.1016/j.fsigen.2013.12.006
97 Walsh, S. *et al.* DNA-based eye colour prediction across Europe with the IrisPlex system. *Forensic Science International: Genetics* **6**, 330-340 (2012). https://doi.org:https://doi.org/10.1016/j.fsigen.2011.07.009
98 Lazaridis, I. *et al.* Genomic insights into the origin of farming in the ancient Near East. *Nature* **536**, 419 (2016). https://doi.org:10.1038/nature19310
99 Mallick, S. *et al.* The Simons Genome Diversity Project: 300 genomes from 142 diverse populations. *Nature* **538**, 201-206 (2016). https://doi.org:10.1038/nature18964
100 Patterson, N., Price, A. L. & Reich, D. Population Structure and Eigenanalysis. *PLOS Genetics* **2**, e190 (2006). https://doi.org:10.1371/journal.pgen.0020190
101 Mathieson, I. *et al.* The genomic history of southeastern Europe. *Nature* **555**, 197 (2018). https://doi.org:10.1038/nature25778
102 Lazaridis, I. *et al.* Ancient human genomes suggest three ancestral populations for present-day Europeans. *Nature* **513**, 409-413 (2014).
103 Villalba-Mouco, V. *et al.* Survival of Late Pleistocene Hunter-Gatherer Ancestry in the Iberian Peninsula. *Current Biology* **29**, 1169–1177 (2019).
104 Brunel, S. *et al.* Ancient genomes from present-day France unveil 7,000 years of its demographic history. *PNAS* **117**, 12791-12798 (2020). https://doi.org:10.1073/pnas.1918034117
105 Chintalapati, M., Patterson, N. & Moorjani, P. The spatio-temporal patterns of major human admixture events during the European Holocene. *eLife* **11**, e77625 (2022).
106 Fenner, J. N. Cross-cultural estimation of the human generation interval for use in genetics-based population divergence studies. *American Journal of Physical Anthropology: The Official Publication of the American Association of Physical Anthropologists* **128**, 415-423 (2005).
107 Fu, Q. *et al.* An early modern human from Romania with a recent Neanderthal ancestor. *Nature* **524**, 216 (2015). https://doi.org:10.1038/nature14558
108 Le Roy, M., Rottier, S. & Tillier, A.-m. Who was a 'Child' During the Neolithic in France? *Childhood in the Past* **11**, 69-84 (2018). https://doi.org:10.1080/17585716.2018.1495141

109 Demirjian, A., Goldstein, H. & Tanner, J. M. A new system of dental age assessment. *Human biology*, 211-227 (1973).
110 AlQahtani, S. J., Hector, M. P. & Liversidge, H. M. Brief communication: the London atlas of human tooth development and eruption. *American Journal of physical anthropology* **142**, 481-490 (2010).
111 Rey, L. *et al.* Tracking male vs. female Neolithic behaviors: a new multi-element and multi-isotope-ratio analysis to reconstruct diet and mobility in northern France. *8th World Archaeological Congress* (2016).
112 Tacail, T., Kovačiková, L., Brůžek, J. & Balter, V. Spatial distribution of trace element Ca-normalized ratios in primary and permanent human tooth enamel. *Science of the Total Environment* **603**, 308-318 (2017).
113 Tacail, T., Télouk, P. & Balter, V. Precise analysis of calcium stable isotope variations in biological apatites using laser ablation MC-ICPMS. *Journal of Analytical Atomic Spectrometry* **31**, 152-162 (2016).
114 R Development Core Team. *R: A language and environment for statistical computing*. (2021).
115 Willmes, M. *et al.* The IRHUM (Isotopic Reconstruction of Human Migration) database--bioavailable strontium isotope ratios for geochemical fingerprinting in France. *Earth System Science Data Discussions* **6** (2013).
116 Bentley, A. R. Strontium isotopes from the earth to the archaeological skeleton: a review. *Journal of archaeological method and theory* **13**, 135-187 (2006).
117 Willmes, M. *et al.* Mapping of bioavailable strontium isotope ratios in France for archaeological provenance studies. *Applied Geochemistry* **90**, 75-86 (2018).
118 Brazier, J. M. *et al.* Determination of Radiogenic 87Sr/86Sr and Stable δ88/86SrSRM987 Isotope Values of Thirteen Mineral, Vegetal and Animal Reference Materials by DS-TIMS. *Geostandards and geoanalytical research* **44**, 331-348 (2020).
119 Horstwood, M., Evans, J. & Montgomery, J. Determination of Sr isotopes in calcium phosphates using laser ablation inductively coupled plasma mass spectrometry and their application to archaeological tooth enamel. *Geochimica et Cosmochimica Acta* **72**, 5659-5674 (2008).
120 Simonetti, A., Buzon, M. R. & Creaser, R. A. In-situ elemental and Sr isotope investigation of human tooth enamel by laser ablation-(MC)-ICP-MS: Successes and pitfalls. *Archaeometry* **50**, 371-385 (2008).
121 Müller, W. & Anczkiewicz, R. Accuracy of laser-ablation (LA)-MC-ICPMS Sr isotope analysis of (bio) apatite–a problem reassessed. *Journal of Analytical Atomic Spectrometry* **31**, 259-269 (2016).
122 Rey, L. *et al.* Disentangling diagenetic and biogenic trace elements and Sr radiogenic isotopes in fossil dental enamel using laser ablation analysis. *Chemical Geology* **587**, 120608 (2022).
123 Goude, G. *et al.* New insights on Neolithic food and mobility patterns in Mediterranean coastal populations. *American Journal of Physical Anthropology* **173**, 218-235 (2020). https://doi.org:org/10.1002/ajpa.24089
124 Redlands, C. A. ArcGIS desktop: release 10. *Environmental Systems Research Institute: Redlands, CA, USA* (2011).
125 Levine, N. CrimeStat III: A Spatial Statistics Program for the analysis of crime incident locations (Version 3.3). Houston, TX: Ned Levine & Associates. (2010).
126 Zaninetti, J. M. *Statistique spatiale. Méthodes et applications géomatiques.*, (Lavoisier, 2005).

127 Le Roy, M. Changes in funerary behaviors from middle to recent Neolithic in France: Evidence from two settlements from the Paris basin. *Archaeologische Korrespondantzblats* (2014).
128 Zvelebil, M. & Pettitt, P. Biosocial archaeology of the Early Neolithic: Synthetic analyses of a human skeletal population from the LBK cemetery of Vedrovice, Czech Republic. *Journal of Anthropological Archaeology* **32**, 313-329 (2013). https://doi.org:10.1017/S0003598X00068307
129 Oksanen, J. *et al.* Package 'vegan'. *Community ecology package, version* **2**, 1-295 (2013).
130 Venables, W. R. & Ripley, B. BD (2002). Modern Applied Statistics with S. *New York: Springer Science & Business Media* **200**, 183-206 (2002).
131 Kuznetsova, A., Brockhoff, P. B. & Christensen, R. H. B. lmerTest package: tests in linear mixed effects models. *Journal of statistical software* **82**, 1-26 (2017).
132 Lenth, R., Singmann, H., Love, J., Buerkner, P. & Herve, M. Emmeans: Estimated marginal means, aka least-squares means. *R package version* **1**, 3 (2018).
133 Reimer, P. J. *et al.* The IntCal20 Northern Hemisphere radiocarbon age calibration curve (0–55 cal kBP). *Radiocarbon* **62**, 725-757 (2020).
134 Sedig, J. W., Olalde, I., Patterson, N., Harney, É. & Reich, D. Combining ancient DNA and radiocarbon dating data to increase chronological accuracy. *Journal of Archaeological Science* **133**, 105452 (2021). https://doi.org:https://doi.org/10.1016/j.jas.2021.105452
135 Mittnik, A. *et al.* Kinship-based social inequality in Bronze Age Europe. *Science* **366**, 731 (2019). https://doi.org:10.1126/science.aax6219
136 Buck, C. E., Kenworthy, J. B., Litton, C. D. & Smith, A. F. Combining archaeological and radiocarbon information: a Bayesian approach to calibration. *Antiquity* **65**, 808-821 (1991).
137 Lanos, P. & Dufresne, P. ChronoModel version 2.0: Software for Chronological Modelling of Archaeological Data using Bayesian Statistics. (2019).
138 Lanos, P. & Philippe, A. Event model: a robust Bayesian tool for chrono-logical model. *HAL* **01241720** (2015).
139 Howell, N. Toward a uniformitarian theory of human paleodemography. *Journal of Human Evolution* **5**, 25-40 (1976). https://doi.org:https://doi.org/10.1016/0047-2484(76)90097-X
140 Lancy, D. F. *The anthropology of childhood: Cherubs, chattel, changelings*. (Cambridge University Press, 2014).
141 Firth, R. *Elements of Social Organisation*. (Watts & Co., London, 1951).
142 Lévi-Strauss, C. *The elementary structures of kinship*. (Beacon Press, 1971).
143 Sellier, P. Tous les morts? Regroupement et sélection des inhumés: les deux pôles du" recrutement funéraire". in *Le regroupement des morts. Genèse et diversité archéologique* (eds Dominique Castex *et al.*) 83–94 (Ausonius Éditions-Maison des Sciences de l'Homme d'Aquitaine, 2011).
144 Ledermann, S. *Nouvelles tables-types de mortalité*. Vol. 53 (Presses universitaires de France, Institut national d'Etudes Démographiques, 1969).
145 Acsádi, G. & Nemeskéri, J. *History of human life span and mortality*. (Akademiai kiado Budapest, 1970).
146 Goude, G. & Fontugne, M. Carbon and nitrogen isotopic variability in bone collagen during the Neolithic period: influence of environmental factors and diet. *Journal of Archaeological Science* **70**, 117-131 (2016).
147 Goude, G., Dori, I., Sparacello, V. S., Starnini, E. & Varalli, A. Multi-proxy dentine microsections analyses reveal diachronic changes in life history adaptations, mobility,

and tuberculosis-induced wasting in prehistoric Liguria (Finale Ligure, Italy, northwestern Mediterranean). *Int J Paleopathol* **28**, 99-111 (2020).
148 Berbesque, J. C., Wood, B. M., Crittenden, A. N., Mabulla, A. & Marlowe, F. W. Eat first, share later: Hadza hunter–gatherer men consume more while foraging than in central places. *Evolution and Human Behavior* **37**, 281-286 (2016).
149 Waguespack, N. M. The organization of male and female labor in foraging societies: Implications for early Paleoindian archaeology. *American Anthropologist* **107**, 666-676 (2005).
