# Supplementary material for: Extensive pedigrees reveal the social organization of a Neolithic community
Source: Nature. 2023 Jul 26;620(7974):600–6. doi: 10.1038/s41586-023-06350-8 (PMC10432279; doi:10.1038/s41586-023-06350-8)
Supplement: Supplementary file 2 — Reporting Summary [file 41586_2023_6350_MOESM2_ESM.pdf]

nature portfolio

Corresponding author(s): Maite Rivollat
Stephane Rottier
Wolfgang Haak

Last updated by author(s): May 3, 2023

Reporting Summary

Nature Portfolio wishes to improve the reproducibility of the work that we publish. This form provides structure for consistency and transparency in reporting. For further information on Nature Portfolio policies, see our Editorial Policies and the Editorial Policy Checklist.

Statistics

For all statistical analyses, confirm that the following items are present in the figure legend, table legend, main text, or Methods section.

n/a	Confirmed	
☐	☒	The exact sample size (*n*) for each experimental group/condition, given as a discrete number and unit of measurement
☐	☒	A statement on whether measurements were taken from distinct samples or whether the same sample was measured repeatedly
☐	☒	The statistical test(s) used AND whether they are one- or two-sided<br>*Only common tests should be described solely by name; describe more complex techniques in the Methods section.*
☒	☐	A description of all covariates tested
☒	☐	A description of any assumptions or corrections, such as tests of normality and adjustment for multiple comparisons
☐	☒	A full description of the statistical parameters including central tendency (e.g. means) or other basic estimates (e.g. regression coefficient) AND variation (e.g. standard deviation) or associated estimates of uncertainty (e.g. confidence intervals)
☐	☒	For null hypothesis testing, the test statistic (e.g. *F*, *t*, *r*) with confidence intervals, effect sizes, degrees of freedom and *P* value noted<br>*Give P values as exact values whenever suitable.*
☐	☒	For Bayesian analysis, information on the choice of priors and Markov chain Monte Carlo settings
☒	☐	For hierarchical and complex designs, identification of the appropriate level for tests and full reporting of outcomes
☒	☐	Estimates of effect sizes (e.g. Cohen's *d*, Pearson's *r*), indicating how they were calculated

Our web collection on statistics for biologists contains articles on many of the points above.

Software and code

Policy information about availability of computer code

Data collection	No specific software was used for data collection.<br>The processing steps from raw Illumina sequence data to genotype data involved the following software:<br>fastqc (0.11.4), EAGER v1 (1.92.56), AdapterRemoval (v2.3.0), BWA (v 0.7.12), DeDup (v 0.12.1), MapDamage (v 2.0.6), samtools (v 1.3.1), pileupCaller (v 1.4.0.2), and bamUtils (v 1.0.13).
Data analysis	ANGSD (0.910)<br>ContamMix (v1.0.10)<br>ADMIXTOOLS (5.1) (qp3Pop, qpDstats, qpAdm)<br>Haplogrep 2 (v2.4.0)<br>Picard tools (v2.27.3)<br>GLIMPSE (v1.1.1) (https://odelaneau.github.io/GLIMPSE/index.html)<br>AncIBD (v0.2a) (https://pypi.org/project/ancIBD/)<br>hapROH (v0.60)<br>Geneious (R8.1.974)<br>READ (v.3) (https://bitbucket.org/tguenther/read)<br>lcMLkin (v0.5.0) (https://github.com/COMBINE-lab/maximum-likelihood-relatedness-estimation)<br>BREADR (v.1.0.1) (https://github.com/jonotuke/BREADR)<br>smartpca (v10210; EIGENSOFT v6.0.1)<br>qpAdm (v.810)<br>DATES (v 753)<br>ISOGG SNP index (v.14.07)

OptiType (v1.3.2) (https://github.com/FRED-2/OptiType)
Genome Analysis Toolkit (GATK) (v.3.5)
HIris-Plex-S tool (https://hirisplex.erasmusmc.nl)
ArcGIS (v.10.8)
CrimeStat (v.3.3)
ChronoModel (v.2.0.18)
R (v 3.6.2), using the following packages: ggplot2 (v3.3.2), dplyr (v1.0.9), tidyverse (v1.3.2), gplots (v3.0.4), magrittr (v1.5), data.table (v1.13.0), janitor (v2.0.1), maps (v3.3.0), mapdata (v2.3.0).
Python (v2.7.18)
The degree of relatedness for a given pair of individuals is calculated with a custom code available at https://github.com/hringbauer/ibd_gurgy/blob/main/notebooks/tree/read_tree.ipynb.
The code for analysing and visualizing the haplotype sharing (IBD and ROH) is deposited at https://github.com/hringbauer/ibd_gurgy.git.

For manuscripts utilizing custom algorithms or software that are central to the research but not yet described in published literature, software must be made available to editors and reviewers. We strongly encourage code deposition in a community repository (e.g. GitHub). See the Nature Portfolio guidelines for submitting code & software for further information.

Data

Policy information about availability of data

All manuscripts must include a data availability statement. This statement should provide the following information, where applicable:

- Accession codes, unique identifiers, or web links for publicly available datasets
- A description of any restrictions on data availability
- For clinical datasets or third party data, please ensure that the statement adheres to our policy

New genomic sequence data (BAM format) is available at the European Nucleotide Archive (ENA) under project accession number PRJEB61818. Previously published genomic sequence data (BAM format) is available at the ENA under the numbers PRJEB36208 and PRJEB45741.
The Genome Reference Consortium Human Build 37 (GRCh37) is available via the National Center for Biotechnology Information under accession number PRJNA31257. The revised Cambridge reference sequence for the mitochondrial genome is available via the National Center for Biotechnology Information under NCBI Reference Sequence NC 012920.1. Previous published genotype data for ancient individuals was reported by the Reich Lab in the Allen Ancient DNA Resource v50.0 (https://reich.hms.harvard.edu/allen-ancient-dna-resource-aadr-downloadable-genotypes-present-day-and-ancient-dna-data).

Human research participants

Policy information about studies involving human research participants and Sex and Gender in Research.

Reporting on sex and gender	*Use the terms sex (biological attribute) and gender (shaped by social and cultural circumstances) carefully in order to avoid confusing both terms. Indicate if findings apply to only one sex or gender; describe whether sex and gender were considered in study design whether sex and/or gender was determined based on self-reporting or assigned and methods used. Provide in the source data disaggregated sex and gender data where this information has been collected, and consent has been obtained for sharing of individual-level data; provide overall numbers in this Reporting Summary. Please state if this information has not been collected. Report sex- and gender-based analyses where performed, justify reasons for lack of sex- and gender-based analysis.*
Population characteristics	*Describe the covariate-relevant population characteristics of the human research participants (e.g. age, genotypic information, past and current diagnosis and treatment categories). If you filled out the behavioural & social sciences study design questions and have nothing to add here, write "See above."*
Recruitment	*Describe how participants were recruited. Outline any potential self-selection bias or other biases that may be present and how these are likely to impact results.*
Ethics oversight	*Identify the organization(s) that approved the study protocol.*

Note that full information on the approval of the study protocol must also be provided in the manuscript.

Field-specific reporting

Please select the one below that is the best fit for your research. If you are not sure, read the appropriate sections before making your selection.

☒ Life sciences ☐ Behavioural & social sciences ☐ Ecological, evolutionary & environmental sciences

For a reference copy of the document with all sections, see nature.com/documents/nr-reporting-summary-flat.pdf

Life sciences study design

All studies must disclose on these points even when the disclosure is negative.

Sample size	No statistical methods were used to determine ancient DNA sample size a priori. Sample sizes for ancient populations depended solely on the availability of the archaeological human remains and on ancient DNA preservation.

Data exclusions	Data from specimens that showed insufficient levels of ancient DNA content or high levels of DNA contamination were excluded from further analyses.
Replication	We studied unique individuals form past populations and did not perform different experiments, so replication is not applicable.
Randomization	We studied unique individuals form past populations and did not perform different experiments, so randomization is not applicable.
Blinding	We studied unique individuals form past populations and did not perform different experiments, so blinding is not applicable.

Reporting for specific materials, systems and methods

We require information from authors about some types of materials, experimental systems and methods used in many studies. Here, indicate whether each material, system or method listed is relevant to your study. If you are not sure if a list item applies to your research, read the appropriate section before selecting a response.

Materials & experimental systems

n/a	Involved in the study	
☒	☐	Antibodies
☒	☐	Eukaryotic cell lines
☐	☒	Palaeontology and archaeology
☒	☐	Animals and other organisms
☒	☐	Clinical data
☒	☐	Dual use research of concern

Methods

n/a	Involved in the study	
☒	☐	ChIP-seq
☒	☐	Flow cytometry
☒	☐	MRI-based neuroimaging

Palaeontology and Archaeology

Specimen provenance	Specimens come from an excavation supported by the region of Bourgogne, France, and the Service Régional de l'Archéologie de Bourgogne, and were sampled with permission from the Service Régional de l'Archéologie de Bourgogne.
Specimen deposition	The skeletal remains are currently stored at the ostéothèque of PACEA, Pessac, France. Further sampling will require permission from the region of Bourgogne, France, and the Service Régional de l'Archéologie de Bourgogne.
Dating methods	New AMS 14C dates were obtained from ultra-filtrated collagen. Collagen extraction and 14C measurements were carried out at the CEDAD - CEntro di DAtazione e Diagnostica, Salento University, Lecce, Italy, and at the CDRC - Centre de Datation par le RadioCarbone, Lyon 1 University, Lyon, France.

☒ Tick this box to confirm that the raw and calibrated dates are available in the paper or in Supplementary Information.

Ethics oversight	No ethics oversight was required strictly, however we confirm that we followed established ethical guidelines for archaeogenetic research.

Note that full information on the approval of the study protocol must also be provided in the manuscript.
